# Disentangling the coupling between sea ice and tundra productivity in Svalbard

**DOI:** 10.1038/s41598-017-06218-8

**Published:** 2017-08-17

**Authors:** Marc Macias-Fauria, Stein Rune Karlsen, Bruce C. Forbes

**Affiliations:** 1 School of Geography & the Environment, University of Oxford, Oxford, OX1 3QY United Kingdom; 2 Norut Northern Research Institute, NO-9294 Tromsø, Norway; 3 Arctic Centre, University of Lapland, FI-96101 Rovaniemi, Finland

## Abstract

The rapid decline in Arctic sea ice poses urgent questions concerning its ecological effects, such as on tundra terrestrial productivity. However, reported sea ice/terrestrial productivity linkages have seldom been constrained, and the mechanism governing them remains elusive, with a diversity of spatial scales and metrics proposed, at times in contradiction to each other. In this study, we use spatially explicit remotely sensed sea ice concentration and high-resolution terrestrial productivity estimates (Normalised Difference Vegetation Index, *NDVI*) across the Svalbard Archipelago to describe local/sub-regional and large-scale components of sea ice/terrestrial productivity coupling. Whereas the local/sub-regional component is attributed to *sea breeze* (cold air advection from ice-covered ocean onto adjacent land during the growing season), the large-scale component might reflect co-variability of sea ice and tundra productivity due to a common forcing, such as large-scale atmospheric circulation (North Atlantic Oscillation, *NAO*). Our study clarifies the range of mechanisms in sea ice/terrestrial productivity coupling, allowing the generation of testable hypotheses about its past, present, and future dynamics across the Arctic.

## Introduction

The steep decline in Arctic sea ice extent, concentration, and volume observed in recent decades has been linked to changes in tundra productivity^1–7^. However, reported correlations between sea ice dynamics and Arctic terrestrial primary productivity are far from coherent and robust over the Arctic – even in places with recent large sea ice reductions^8^, while the mechanism that drives this coupling remains elusive. Without exception, the Arctic Amplification (hereafter *AA* ^9^) has been implicitly or explicitly inferred to drive the terrestrial productivity/sea ice connection. *AA* consists of a suite of processes operating at northern high latitudes at different temporal and spatial scales^9^ resulting in high rates of temperature variability and strong climate change trends in the Arctic. These have been calculated to be ~1.6–2 times the global average in recent decades using observational climate data^10^, and 3–4 times over longer term – millennia to millions of years – using proxy data^11^.

Positive feedbacks linked with the high albedo and thermal insulation properties of ice and snow constitute a large component of the *AA* ^9, 12^, and are thought to mostly operate in autumn and winter, when the heat stored during the warm sunlit season by the upper ocean mixed layer is released into the atmosphere. Summer is the season with the weakest *AA*, potentially affected by snow albedo early in the season^9^. Processes other than sea ice and snow albedo feedbacks play important roles in the *AA*, especially those related with temperature feedbacks in the vertical structure of the warming (*lapse-rate feedback*) and the relationship between radiative forcing, temperature, and longwave emission (*Planck’s feedback*)^13^, among others^9^. Thus, the effects of an *AA*-enhanced warmer Arctic on terrestrial ecosystem functioning cannot be linked to sea ice decline without previously defining and scaling candidate mechanisms able to effectively couple sea ice and such ecological processes. In the case of tundra productivity, and given its widely reported strong relationship with growing season temperatures^14–16^, we will focus on processes responsible for sea ice-related summer *AA*.

Bekryaev, *et al*.^10^ described large-scale (hemispheric) and local/sub-regional spatial components in *AA*. The former is seen as the amplification of zonally averaged surface air temperature variations from tropical through high-latitude regions, whereas local/sub-regional *AA* was related to ice-albedo feedbacks in maritime and coastal areas in autumn when the ice cover is seasonally at a minimum^10^. A further local/sub-regional manifestation of *AA* was described by Haugen and Brown^17^, termed *sea breeze* and consisting of cold air advection in summer from the partially to fully ice-covered ocean onto the adjacent land. Both local/sub-regional processes can influence temperatures several hundreds of km inland, depending on topography^10, 17^. By definition, large-scale *AA* has a loose spatial resolution and its sea ice component is difficult to quantify without climate modelling^13^ or the study of many climatological variables^18^, especially given the similar recent trends between pan-Arctic sea ice and Arctic temperatures: temperature-sensitive terrestrial ecological processes might thus show high correlations with hemispheric/regional sea ice extent even when this is distant. Local/sub-regional *AA* reflects much more spatially and temporally constrained phenomena: (1) if present, sea ice cools air above it and enhances cold air advection onto adjacent, warmer landmasses (summer), and/or 2) open ocean water releases heat that warms coastal areas (autumn). Local/sub-regional *AA* can effectively decouple surface air temperature from synoptic-scale circulation. The dimension of the coupling mechanism between sea ice and tundra productivity will leave a signature in the spatial patterns of co-variability between both variables and allows the formulation of hypotheses:

- in the local/sub-regional *AA*, spatially-constrained sea ice adjacent to land will be highly related with on-land terrestrial productivity;
- in the large-scale/hemispheric *AA*, distant and less spatially-constrained sea ice will be related to tundra terrestrial productivity.

Here we show evidence for strong large and local/sub-regional scale co-variability between sea ice concentration and tundra terrestrial productivity in the Svalbard Archipelago, demonstrating and scaling the ways in which sea ice might affect terrestrial primary productivity in the Arctic. While we attribute the local/sub-regional component of this coupling to cold air advection (the *sea breeze* mechanism proposed by Haugen and Brown^17^), we show the large-scale component to be mostly related with large-scale/hemispheric atmospheric patterns (North Atlantic Oscillation, *NAO* ^19^) affecting both sea ice and temperatures during the growing season, and causing their co-variability. Svalbard encapsulates much of Arctic’s range of environmental conditions in a relatively small area: whereas the West Spitsbergen Current delivers warm North Atlantic surface water to its western half (hereafter *W-Sb*), which is often free of ice, the East Spitsbergen Current delivers cold, Arctic surface water to the eastern parts of the archipelago^20^ (hereafter *E-Sb*), which are colder and covered by sea ice for much of the year, well into the growing season. Moreover, Svalbard’s rugged topography favours large environmental gradients over short distances, maintaining the contrasting east-west environmental divide driven by ocean surface currents.

Glaciers cover >60% of the total area in the Svalbard archipelago, whereas much of the non-glaciated land is dominated by open, non- or sparsely vegetated landscapes^21^. Vegetated regions concentrate in the lowlands on central and Northern Spitsbergen, Barentsøya, and Edgeøya (Supplementary Material 1). In *W-Sb*, central Spitsbergen shows low glacier coverage and large areas of wetland and bryophyte-dominated vegetation – including moist tussock tundra – in low valleys (>400 km^2^), with a diversity of *Dryas* tundra vegetation on ridges and hillslopes (>650 km^2^), and arctic meadows on south-facing slopes (~150 km^2^). Vegetation in *E-Sb* is concentrated in Barentsøya and Edgeøya, where glaciers cover ~44% of the area: here, vegetation types range from dense *Dryas* vegetation to well-developed mires and bryophyte-dominated vegetation along the eastern shores, to polar desert in the eastern lowlands^21^.

We used spatially explicit, 8-day averaged, remotely sensed data on sea ice concentration (resampled to 2.3 km pixel resolution) and *NDVI* at 0.5 km pixel resolution – widely used as an indicator of terrestrial primary productivity in the Arctic^22–25^, covering the whole archipelago and adjacent ocean waters for the period 2000–2014, and focused on the growing season (defined as June 10^th^ – September 6^th^, see Methods). Singular Value Decomposition (*SVD*)^26–28^ was applied to both sets of data (fields). *SVD* identifies pairs of coupled spatial patterns and their temporal variation – modes – explaining the maximum mean-squared covariance between two spatially separate fields (in this case sea ice and *NDVI*). *SVD* analyses were performed for the whole growing season and for three separate periods – defined as Early Growing Season – *EGS*, Peak Growing Season/July – *JL*, and Late Growing Season – *LGS*, and for *W-Sb* and *E-Sb* (see Methods).

## Results

Coherent patterns of sea ice *vs*. terrestrial productivity coupling emerged from the *SVD* analysis. In all instances, the 1^st^ mode in the *SVD* was found to be significant and to explain >60% of the covariance (ranging from 62.63% to 89.38%), with the percentage of the explained total variance in the two fields ranging from 17.86% to 31.27% (Supplementary Material 2). In some instances, higher-order *SVD* modes were found to be significant, however they explained a much smaller proportion of the covariance and will not be discussed in this study.

**Figure 1:**
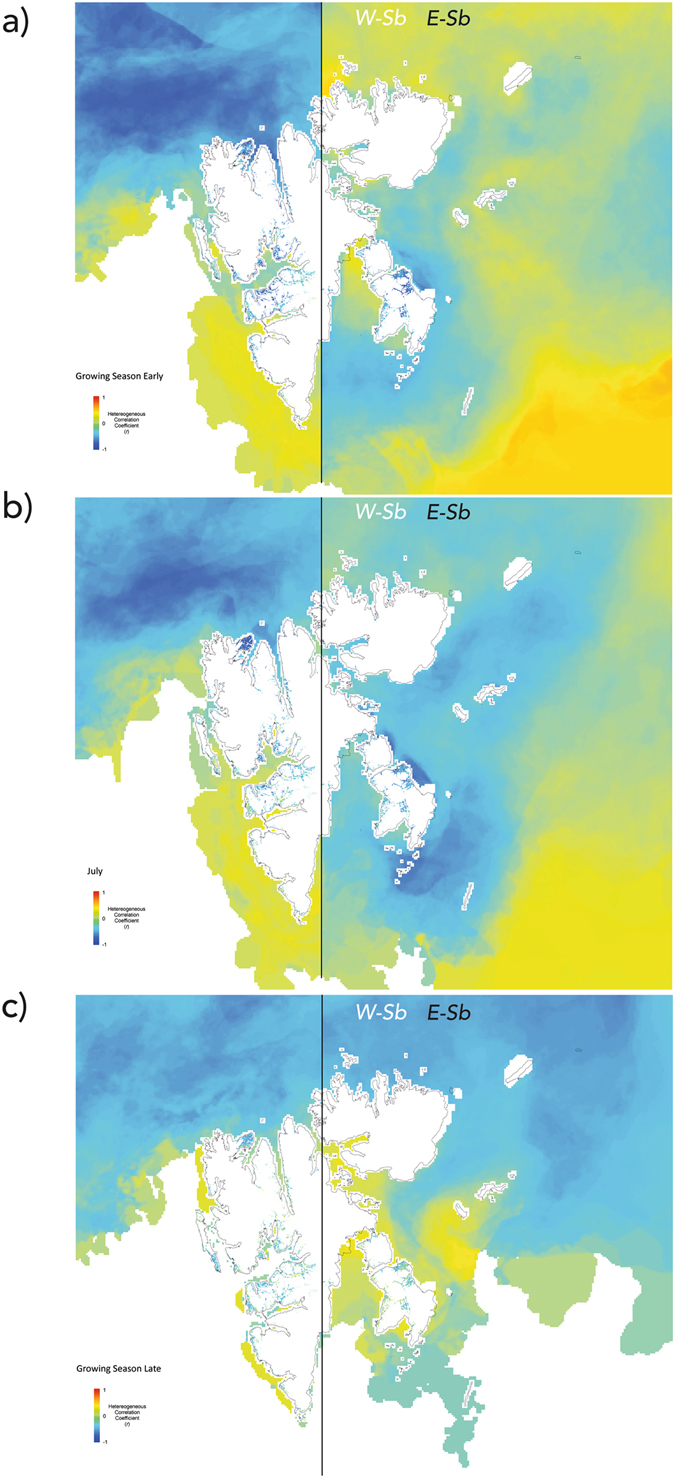
Heterogeneous Correlation Maps. Heterogeneous Correlation Maps between sea ice concentration and Normalised Difference Vegetation Index (*NDVI*) anomalies for the 1^st^ Singular Value (*SV*) during (**a**) the Early Growing Season (*EGS*; June 10 – July 4); (**b**) the Peak of Growing Season (*JL*; June 26 – August 5); and (**c**) the Late Growing Season (*LGS*; August 5 – September 6, see Methods) for the West (*left panel*) and East (*right panel*) halves of the Svalbard Archipelago. Land pixels show the correlation between observed *NDVI* (period 2000–2014) and the expansion coefficients of Sea Ice for the *1* ^*st*^ *SV*; Ocean pixels show the correlation between observed Sea Ice concentration (period 2000–2014) and the expansion coefficients of *NDVI* for the *1* ^*st*^ *SV*. Maps were generated using Matlab (version R2016b; https://www.mathworks.com) and ArcMap (version 10.2; https://www.esri.com), used herein under license.

Heterogeneous Correlation Maps depict the temporal linear correlation between the expansion coefficients of one field and each grid-point containing a timeseries of observations on the other field – see Methods, indicating how the two fields are related to one another and how much of the amplitude of their variation is explained by the *SVD*, delivering information that hints at the nature of the coupling between the two fields^27^. They showed overall high negative relationships between *NDVI* and the expansion coefficients of sea ice, and sea ice and the expansion coefficients of *NDVI*. The early growing season (*EGS*) in Svalbard was characterised by a sharp difference between its west and east halves (Fig. 1a). In *W-Sb*, *NDVI* corresponding to vegetated land surface was found to be negatively related with sea ice, however the relevant sea ice region was located in the northern Arctic Ocean, >250–300 km from most vegetated land masses: that is, sea ice conditions in the ocean sections adjacent to land were unrelated with *NDVI*, except for northernmost Spitsbergen (Reinsdyrflya, see Fig. 1a, Supplementary Material 1). *E-Sb* depicted a different spatial arrangement: *NDVI* corresponding to vegetated land surface (mostly on Edgeøya, see Fig. 1a, Supplementary Material 1) was also strongly and negatively related with sea ice, however the ocean areas involved in this spatio-temporal coupling were located adjacent to the land (<50 km). Sea ice conditions further away were unrelated to the dynamics of *NDVI* in *E-Sb*. Similar and enhanced patterns emerged in both regions during the month of July (*JL*; Fig. 1b), at the peak of the growing season. Finally, the coupled spatial patterns in *E-Sb* during the late growing season (*LGS*) shifted to an arrangement resembling that of *W-Sb*, with strong paired coupling between *NDVI* in vegetated land masses and sea ice concentration anomalies in distant northern ocean areas, coinciding with the retreat of sea ice from the region in late summer (Fig. 1c; Supplementary Material 3).

Sea ice concentration was found to be highly (and negatively) related with the *NAO* index in *W-Sb* (peaking in July, when mean sea ice concentrations for the ocean area in *W-Sb* correlated at *r* ^*2*^ = 0.58 with mean *NDVI* values for the same period over the land areas in *W-Sb*). Moreover, the spatial arrangement of *NAO vs*. sea ice concentration mimicked that of the heterogeneous correlations between sea ice and *NDVI* (Fig. 1; Supplementary Material 4). Whereas *NAO* explained some variability in *E-Sb* (peaking in July at *r* ^*2*^ = 0.36, as did the Arctic Oscillation, *AO* − *r* ^*2*^ = 0.36), the spatial arrangement of *NAO* & *AO vs*. sea ice concentration did not agree with that of the heterogeneous correlations between sea ice and *NDVI* (Fig. 1; Supplementary Material 4). Moreover, only *W-Sb* sea ice concentration showed field significance with *NAO* (in July, p < 0.01 – see Methods). *NDVI vs. NAO* showed field significance for *W-Sb* June through August, as did *NDVI vs. AO* in June and July in *W-Sb* and in July only in *E-Sb* (Supplementary Material 4).

The above-mentioned *NDVI vs. NAO* patterns of spatio-temporal co-variability are in agreement with the generalised positive correlation between monthly *NAO* and surface air temperatures from the same period 2000–2014 obtained from the NCEP-NCAR Reanalysis project^29^ (Supplementary Material 5; see Methods), also peaking in July, especially in the southern half of the archipelago. Sea ice *vs. NAO* negative correlation patterns also agree with the positive relationship between *NAO* and meridional wind (Supplementary Material 5; see Methods), being especially strong in *W-Sb* in July. *AO vs*. meridional wind showed weak relationships, in line with the observed non-significant field correlations between sea ice and *AO*, whereas mostly positive temperature/*AO* relationships occurred in July (Supplementary Material 5; see Methods).

**Figure 2:**
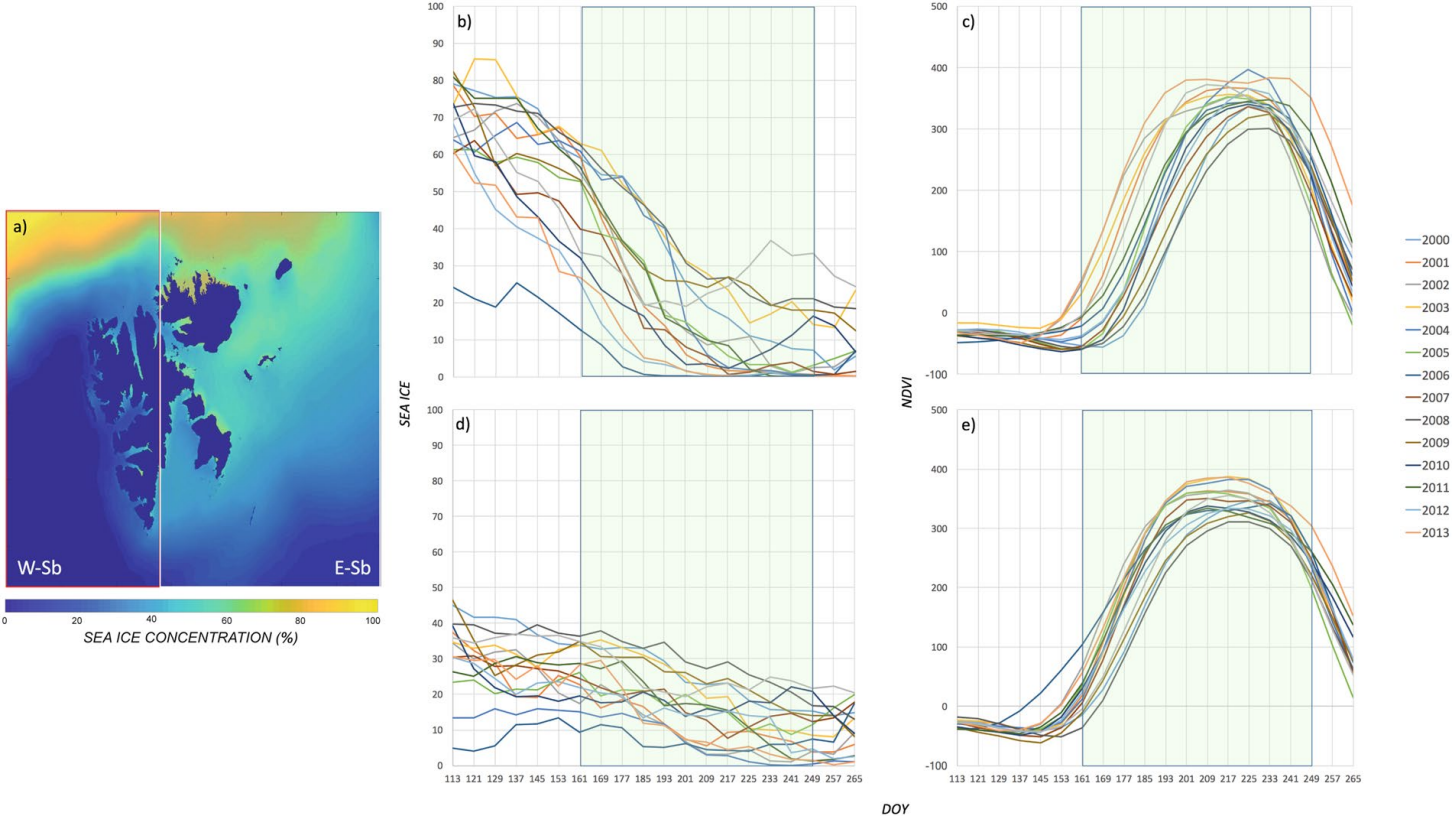
Sea ice and NDVI variability in *W-* & *E-Sb*. (**a**) Map of the Svalbard Archipelago depicting mean sea ice concentration for the period 2000–2014 and the extent of *W-Sb* & *E-Sb*. Eight-day mean sea ice concentration and *NDVI* values are shown for *E-Sb* **(b**,**c**) and *W-Sb* (**d**,**e**) for each year from 2000 to 2014 during the period of the year for which good quality data is available (Day-of-Year, DOY 113 – April 23 to DOY 273 – September 30). The growing season (defined here as DOY 161 to DOY 249 – see Methods) is highlighted as a green box. Note the larger inter-annual variability in the early growing season starting date and phenology as seen in the *NDVI* values in *E-Sb*, and the overall larger sea ice concentration in *E-Sb* during the early and peak of the growing season, which reaches similar values to those in *W-Sb* in the late growing season only (~DOY 209) onwards. The map in Fig. 2a was generated using Matlab (version R2016b; https://www.mathworks.com), used herein under license.

The different dynamics of the sea ice *vs. NDVI* coupling between *W-Sb* and *E-Sb* are further exemplified by their different seasonality (Fig. 2). *E-Sb* showed generalised high sea ice concentrations during the growing season (Fig. 2b) and a large inter-annual variability in the *NDVI* seasonality (Fig. 2c). Conversely, *W-Sb* showed low values of sea ice concentration during the growing season (most of this ice being in the far north of the region, away from vegetated areas; Fig. 2c) and a much lower inter-annual variability in the seasonality of *NDVI* (Fig. 2d). The time difference in the start of the growing season between an early-starting and a late-starting year was >30 days in *E-Sb* and ~16 days in *W-Sb* (Fig. 2c,e). *E-Sb* sea ice concentration values became similar to those in *W-Sb* late in the growing season (Fig. 2b,d; Supplementary Material 3).

## Discussion

The quest for understanding the link between tundra terrestrial productivity and sea ice dynamics is embedded in the urgent need to describe the ways in which ecological systems are coupled with marine and atmospheric physical processes in a time of accelerated changes in the Arctic. The use of a spatially explicit approach employing remote sensing observations of sea ice and tundra productivity enabled the formulation of hypotheses based on the expected spatial patterns that should result from sea ice *vs*. tundra productivity coupling mechanisms operating at different scales. The observed paired modes of variability in *E-Sb* clearly suggest that in the presence of abundant sea ice close to tundra-covered land during the growing season, *sea breeze* ^17^ effectively advects cold air over adjoining coastal lands, lowering local/sub-regional air temperatures and negatively affecting plant productivity. Moreover, the patterns of spatiotemporal coupling in *E-Sb* in Fig. 1a,b further favour local sea ice presence as a driving mechanism for *NDVI* dynamics, since most of the ocean areas in *E-Sb* are moderately to abundantly covered with sea ice during the growing season and yet only the regions closest to the vegetated Edgeøya and Barentsøya show high agreement with tundra productivity dynamics. The sharp change in the spatial arrangement of the paired co-variability structures in the late growing season (Fig. 1c) and their shift towards the type of dominant pattern in *W-Sb* coincide with the seasonal disappearance of sea ice in the area and the convergence of sea ice spatial configuration with *W-Sb* (Fig. 2b,d; Supplementary Material 3). This suggests that in the *LGS* sea ice has dropped to levels where it does not affect adjacent land productivity via *sea breeze*. Alternatively, it may suggest that temperature-unrelated processes such as photoperiod might dominate terrestrial productivity during the LGS^8^. Finally, *SVD* statistics point towards a strong coupling between sea ice and *NDVI*, since they are very high compared to other *SVD*-based studies^26, 27^ (Supplementary Material 2).

In contrast, *NDVI* in *W-Sb* is in consistent high agreement with sea ice concentration anomalies in ocean areas located at the northernmost region of the study area, very distant from most of the tundra-covered regions in Spitsbergen (notably the central large fjord systems in the interior and west of the island, >250–300 km away from the relevant sea ice region), except for the northernmost Reinsdyrflya peninsula. Sea ice concentration in areas closer to these inner fjords was not found to affect tundra productivity, perhaps unsurprisingly given the fact that during the growing season there is very little sea ice in the central and south ocean regions of *W-Sb*. The relationships between sea ice and *NAO*, *NAO* and meridional wind, *NAO* and *NDVI*, and *NAO* and surface air temperatures are strong in *W-Sb* during the period analysed – and mostly in *JL*. *NAO* has long been known to play a role in the North Atlantic storm track and thus in the advection of warmer water and air masses into the Barents Sea through the West Spitsbergen Current, affecting the position of the sea ice edge in the region^30, 31^. The flow of warm water and air advection associated with *NAO* can thus affect both sea ice and tundra productivity without them needing to be coupled mechanistically. Hence, the existence of a correspondence between sea ice and *NDVI* anomalies in this region (and in *E-Sb* during the *LGS*) may be due to the influence of a *lurking variable* or ultimate process driving both. Finally, the relationships of *NAO* and *AO vs*. sea ice and *NDVI* in *E-Sb* for *JL* showed different spatial patterns to those in *W-Sb*: the lack of correspondence between the *NAO*/*AO* correlation fields and the *SVD* paired patterns of co-variability suggests that the role of these teleconnection modes is not linked with the local/sub-regional *NDVI*/sea ice coupling captured by the *SVD* in *E-Sb*. The fact that only *NDVI* – and not sea ice concentration – showed field significance with *AO* and *NAO* (July, p < 0.05) in *E-Sb* further suggests that this mechanism might not be led by sea ice (Supplementary Material 4).

The local/sub-regional *vs*. large-scale nature of the driving mechanisms in *W-Sb* and *E-Sb* can also be seen in the seasonal dynamics of sea ice and *NDVI* (Fig. 2). Whereas large sea ice concentration and *NDVI* variability are seen in *E-Sb* during *EGS* and *JL*, variability in sea ice concentration does not translate in variability of *NDVI* seasonality in *W-Sb*. This agrees with the much higher sea ice concentration in *E-Sb* enabling effective *sea breeze* to operate up to the *LGS*. The smoother and more synchronous dynamics of *NDVI* in *W-Sb* agree with a less spatially constrained controlling mechanism driving terrestrial vegetation growing season. Although the lack of meteorological records covering the study period in *E-Sb* precluded us from analysing the relative variability of temperatures during the growing season between *W-Sb* and *E-Sb* and the influence of local sea ice on surface air temperatures, records exist from 12 meteorological stations – 6 in *W-Sb* and 6 in *E-Sb* – for the 3-year period 2012–2014. Over this shorter period, *E-Sb GS* 8-day mean surface air temperatures were overall colder than *W-Sb* stations, and displayed both significantly higher inter-station and year-to-year intra-station variability (Supplementary Material 6 – see Methods), in line with a suggested more localised mechanism controlling temperature. This data-limited results strongly encourage the immediate undertaking of research efforts directed at obtaining high-resolution, spatially explicit, remotely-sensed temperature records for the archipelago to provide a more comprehensive picture of the suggested mechanism that includes the spatio-temporal coupling between air temperatures and sea ice.

The local/sub-regional *AA* mechanism gives clear and unequivocal spatial signatures, however the spatial signature of the large-scale mode of co-variability is far from unequivocal: whereas we show evidence to suggest that a *lurking variable* (in this case large-scale atmospheric circulation) affects both sea ice concentration and *NDVI*, a possible influence of sea ice on the regional temperatures cannot be completely ruled out. The coupling between sea ice dynamics and the climate system is not unidirectional and changes in sea ice might feedback to the climate system over a wide range of spatial and temporal scales, affecting not only the Arctic but the Northern Hemisphere mid-latitudes through their influence on storm tracks, the jet stream, and planetary waves and their associated energy propagation^32, 33^, as well as the *NAO* ^34^. Such evidence – stilly bound by large uncertainties^33^ – further complicates the interpretation, understanding, and quantification of the large-scale linkages between sea ice and tundra productivity, which require more than correlational studies between sea ice and tundra productivity estimates, involving the employment of regional climate and coupled sea ice/climate models, in which sea ice forcing on climate is quantified in the same manner as in the [CO_2_]_atmosphere_ attribution exercises^35, 36^.

The framework presented herein can characterise candidate mechanisms driving the patterns reported elsewhere on sea ice *vs*. tundra productivity. Local/sub-regional summer *AA* – *sea breeze* – agrees with the findings by Dutrieux, *et al*.^7^ in a set of transects in the Siberian Arctic, who found a decreasing strength in the sea ice concentration *vs*. summer warmth index (*SWI*) and time-integrated *NDVI* (TI-*NDVI*) correlations with distance to the coast (however with weak values). The same mechanism can be applied to Macias-Fauria, *et al*.^8^ in Yamal and Nenets Autonomous Okrugs, who found sea ice in the Barents and Kara Seas to be only correlated with *NDVI* in May and argued that during the growing season sea ice might be too distant from the tundra to affect productivity dynamics. Hence, the presented local/sub-regional *sea breeze* is a strong candidate to operate over the whole Arctic and its spatial signature may be found elsewhere provided that sea ice is close to vegetated land during the growing season.

Reported coarser-scale/pan-Arctic relationships^1, 2, 4^ are less well constrained and potentially more complex: in these cases, large-scale mechanisms might be at play, which would require further analyses prior to a robust sea ice attribution on tundra dynamics^3^. The identified *NAO* (and to a lesser degree *AO*) large-scale linkage in the case of the Svalbard Archipelago might not be operating in the same way elsewhere in the Arctic. Further, studies reporting large-scale sea ice/tundra productivity relationships are often based on summary statistics (e.g. TI-*NDVI*) whose signal is likely compounded as it incorporates and averages several processes and events over the growing season. Kerby and Post^5^ reported strong relationships between winter and June pan-Arctic sea ice extent and the phenology of several tundra species in Kangerlussuaq (west Greenland). The poor explanatory power of local sea ice and summer temperatures in this study suggests a minor role played by local/sub-regional *AA*. In this case, the already challenging task of quantifying the influence of large-scale sea ice variability on climate is compounded by the unknown suite of climatic conditions that might be controlling plant phenology and which pan-Arctic sea ice extent might integrate^5^. Relationships between plant productivity and sea ice outside of the growing season^5, 6^ represent a further nuance: Hollesen, *et al*.^6^ – working in Disko Island, <300 km north of Kangerlussuaq – proposed a mechanism through which local winter sea ice conditions might be coupled with local winter temperatures, which are related with increased radial growth of *Betula nana* through earlier soil warming. In this case, the nature of the coupling might be due to (1) local coupling or (2) collinearity between winter air temperatures and local sea ice extent driven by a third process (e.g. ocean circulation). Although we focused on temperature as it is the main variable controlling terrestrial productivity in the Arctic and in Svalbard in particular^14–16^, other climate variables might be important locally or regionally, such as precipitation^15^: in this cases, the effects of sea ice on Arctic precipitation^37, 38^ will likely explain a significant amount of tundra productivity variability. Finally, sea ice/tundra productivity coupling might also incorporate long-term, low-frequency processes that are out of the scope of this 15-year study.

Knowledge of the mechanism allows for the generation of predictions and hypotheses: year-round current and projected future sea ice decline^39^ implies sea ice retreat from tundra-covered land masses earlier in the year, increasing the tundra areas that might become decoupled from the local/sub-regional *AA* and (1) increasingly behave like *W-Sb* or (2) stop showing any local/sub-regional relationship with sea ice: *E-Sb*-like areas might have recently transitioned or will soon transition into *W-Sb*-like ones. Although the dynamics of *sea breeze* without sea ice may include further feedback mechanisms^3^, our results strongly suggest that once sea ice is absent from the ocean adjacent to tundra regions, *sea breeze* does not significantly affect tundra productivity (Fig. 1)^8^. In contrast, research is still needed to understand and robustly forecast likely future tundra *vs*. sea ice coupling under the large-scale *AA* mechanism(s). In these cases, predictions and observations are still in a *black-box stage* and are thus extremely sensitive to training data.

## Conclusions

In this study, we showed that distinctly paired sea ice/tundra productivity spatial signatures result depending on the scale and characteristics of the dominant coupling mechanism. We clearly identified local/sub-regional and large-scale modes of variability in the Svalbard Archipelago which are geographically robust and which can be explained by the spatial arrangement of sea ice in the ocean areas surrounding tundra-covered land masses. Presence of abundant sea ice near the coast during the growing season favours local control of tundra productivity by sea ice, very likely through *sea breeze* ^17^. The large-scale paired pattern of co-variability is clearly identified and dominates regions where sea ice is distant from vegetated land masses. In this case, we advise against attributing tundra dynamics to sea ice based solely on correlation between both fields: in our study area in Svalbard, we present evidence that supports large-scale atmospheric and sea surface dynamics (captured by the *NAO* index) affecting both sea ice and growing season temperatures and thus causing high agreement between both fields without the need of a mechanistic link. Attribution studies and/or the detailed analyses of many more climatological variables would be needed in this case to quantify the relative effect of sea ice on tundra productivity.

Our study (1) provides a framework to investigate the potential ways in which the sea ice/tundra productivity coupling takes place through remote sensing; (2) improves the understanding of these mechanisms, refining their spatial dimension; and (3) generates testable hypotheses for sea ice/tundra productivity dynamics in the Arctic, both at present and in past and future scenarios, which might involve spatiotemporal shifts between local/sub-regional and large-scale controls.

## Material and Methods

### Data

Sea ice concentration data for the ocean areas surrounding Svalbard were obtained from the Norwegian Sea Ice Service (http://wms.met.no/icechart/). The dataset has close to daily time resolution and is based on the best possible satellite data available as found from visual inspection. In our study area, up to 100 m resolution Synthetic Aperture Radar – SAR – data, and sometimes passive microwave data at ~25 km resolution were used. If cloud free, NOAA-AVHRR data was employed, and in only very few cases (<100 days in our study area/period, comprising ~2500 days in total) Moderate Resolution Imaging Spectroradiometer (MODIS) imagery was included. The dataset was converted to raster and consists of mean 8-day sea ice concentration values for the period 2000–2014. Sea ice concentration was categorised into six discrete classes, as recommended by the Norwegian Sea Ice Service: 5% – Open Water (0/10); 25% – Very Open Drift Ice (1/10 to 4/10), 55% – Open Drift Ice (4/10 to 7/10), 80% – Close Drift Ice (7/10–9/10), 90% – Very Close Drift Ice (9/10–10/10), 100% – Fast Ice (10/10). The spatial resolution of the sea ice data was 2,320 m * 2,320 m.

Eight-day mean clear-sky Normalised Difference Vegetation Index (*NDVI*) data was obtained from MODIS^40^. To process a clear-sky *NDVI* dataset, we used the MODIS Terra data surface reflectance products MOD09Q1 and MOD09A1, both with 8-day temporal composites. Due to the short and intense period of plant growth in the High Arctic and the occurrence of frequent cloud cover, all possible cloud free data is needed, which requires reliable cloud masks to be computed. A combination of three cloud-removing methods was used^40^. The quality assessment (QA) flags in the MOD09A1 product give information about different types of atmospheric noise. For each 8-day period, we made a visual quality control in ArcGIS of which of the different State QA values detected the cloud cover and cloud shadows. In most cases (>60%), one of the State QA values or a combination of the State QA values could detect the clouds in the data. This approach was not sufficient in the remaining <40% of the cases, depending on the cloud type, time of the season, and type of land cover, and especially in the detection of cloud shadows and clouds over sparsely vegetated areas. Therefore, we developed our own cloud detection algorithms based on the 7 bands in the MOD09A1 products^40^. In the few cases where both algorithms failed, we manually masked out the noisy parts by drawing polygons around them. Altogether, 15 different combinations of State QA values/own algorithms/manual masking were employed in the cloud detection and removal procedures. For each of these combinations, a python script to remove the clouds was developed. The cloud mask was then applied to the *NDVI* values calculated form the MOD09Q1 product. Savitzky-Golay spatial interpolation was applied to pixels and periods with no available data. The spatial resolution of the *NDVI* data was resampled to 464 m * 464 m.

The growing season (*GS*) was defined as the period encompassing Day-of-Year (DOY) 161 (June 10) to 249 (September 6); that is, eleven 8-day periods, forming a time series of 165 observations over the 15-year period. To study the potential change of the paired spatial patterns as sea ice recedes during summer, *GS* was divided into 3 sub-periods: Early Growing Season (*EGS*), DOY 161 (June 10) to 185 (July 4), consisting of three 8-day periods forming a time series of 45 observations over the 15-year period; Peak Growing Season, spanning mostly the month of July (*JL*), DOY 177 (June 26) to 217 (August 5), consisting of five 8-day periods forming a time series of 75 observations over the 15-year period; and Late Growing Season (*LGS*), DOY 217 (August 5) to 249 (September 6), consisting of four 8-day periods forming a time series of 60 observations over the 15-year period.

The study area was divided in two halves, named West Svalbard – *W-Sb* – and East Svalbard – *E-Sb*, to study the dynamics of the coupling between sea ice and terrestrial tundra productivity in a region with overall low sea ice concentration (*W-Sb*) and in one with overall high sea ice concentration (*E-Sb*) during the growing season (e.g. Fig. 2a).

### Singular Value Decomposition

The *Singular Value Decomposition* analysis has been widely used in climatology to find relationships between two spatial fields^26–28^ (in this study sea ice concentration and *NDVI*) by decomposing the covariance matrix of the two fields into singular values and two sets of paired orthogonal vectors, one for each field. *SVD* maximises the covariance between the expansion coefficients of the leading pattern in each field^27^, identifying pairs of spatial patterns that explain as much as possible of the mean-squared temporal covariance between them. Iwasaka and Wallace^27^ explain in detail the *SVD* and the methodology described in here is largely based on their work.

*SVD* was computed over (1) the whole study region and for the *GS*, (2) for *W-Sb* and *E-Sb* separately, and (3) for *EGS*, *JL*, and *LGS* for the whole region, *W-Sb*, and *E-Sb*. We first constructed anomaly datasets from the raw sea ice and *NDVI* data by subtracting from the 8-day values their climatological mean, and normalised them by dividing this value by the standard deviation of the 8-day period. This resulted in a Sea Ice Anomaly field (***S***) and an NDVI anomaly field (***P***). For the *GS*, ***S*** consisted of a 165 * 80,555 matrix (15 years * eleven 8-day periods per year, 80,555 pixels with sea ice concentration data in them), whereas ***P*** was a 165 * 12,774 matrix. ***S*** and ***P*** had 45 rows for *EGS*, 75 rows for *JL*, and 60 rows for *LGS*. Matrices for *W-Sb* and *E-Sb* consisted of the same number of rows (i.e. they were computed over the same period) but fewer pixels each (***S*** & ***P*** for *W-Sb* were 31,507 & 9,212 columns, respectively, and ***S*** & ***P*** for *E-Sb* were 49,048 & 3,532 columns, respectively).

Next, the cross-covariance matrix was computed, ***C*** = ***S***′ * ***P***/***N***, where ***N*** = length of the period. ***C*** was then used to compute the *SVD*. *SVD* decomposes ***C*** in two matrices of singular vectors: ***U*** – for sea ice, and ***V*** – for *NDVI*). Each singular vector is normalised and non-dimensional, and each pair of singular vectors is a mode of co-variability between the fields ***S*** & ***P*** (e.g. the two first singular vectors in ***U*** and ***V*** represent the spatial patterns that explain most of the covariance between ***S*** & ***P***). From the singular vectors and the fields, the expansion coefficients can be computed (***A*** = ***S*** * ***U***, expansion coefficients of sea ice; ***B*** = ***P*** * ***V***, expansion coefficients of *NDVI*), which are time series describing how each mode of variability changes with time, and have been dimensioned to the original data (Supplementary Material 2).

To investigate the strength of the relationship found between two fields, the **squared covariance fraction** – ***SCF*** – measures the relative importance of a given *SVD* mode in the relationship between the two fields and it is computed as ${\boldsymbol{SC}}{{\boldsymbol{F}}}_{{\boldsymbol{k}}}=\frac{{\pi }_{k}^{2}}{{\sum }_{i=1}^{n}{\pi }_{i}^{2}}\ast 100\,( \% )$, where *k* is the mode and *π*_*k*_ is the singular value and the *k* ^th^ mode. The **normalised root-mean squared covariance fraction** is computed as ${\boldsymbol{NC}}{{\boldsymbol{F}}}_{{\boldsymbol{k}}}={[\frac{{\pi }_{k}^{2}}{{\sum }_{i}{\sum }_{j}{\sigma }_{i}^{2}{\sigma }_{j}^{2}}]}^{\frac{1}{2}}\ast 100\,( \% )$, where σ_i_^2^ and σ_j_^2^ are the variances of the *i*-th grid point of one field and that of the *j*-th grid point of the other field, respectively^27^. ***NCF***_***k***_ gives a measure of the absolute importance of the *SVD* mode in the relationship between the two fields. Further, the correlation between the expansion coefficients (A * ***B***) for each mode – ***ρ***_***k***_ – indicates the similarity between the time variations of the patterns of the two fields. These statistics are shown in Supplementary Material 2.

Statistical significance of the *SVD* modes was computed by estimating confidence intervals on ***SCF*** and ***NCF*** via a Monte-Carlo approach based on 100 runs of the *SVD* for each pair of fields (*GS*, *EGS*, *JL*, and *LGS*), but with one of them being randomly shuffled in the time sequence^27^.

The computation of homogeneous and heterogeneous correlation maps provides important information on the spatial structure of the covariance between the two fields. For each *SVD* mode (***SVD***_***k***_), two expansion coefficient vectors exist (***A***_***k***_ & ***B***_***k***_), one for each field (sea ice and *NDVI*). ***Homogeneous correlation maps*** are computed by correlating the expansion vector for a given mode with the time series corresponding to the same field variable at each grid point (i.e. ***A***_***k***_ is correlated with sea ice, ***B***_***k***_ is correlated with *NDVI*). ***Heterogeneous correlation maps*** are computed correlating the expansion vector of one field for a given mode with the time series corresponding to the other field at each grid point (i.e. ***A***_***k***_ is correlated with *NDVI*, ***B***_***k***_ is correlated with sea ice). The latter show how the two fields are related to one another and how much of the amplitude of their variation is explained by the *SVD* ^27^: although they are expected to be lower than the homogeneous correlation maps, they deliver very important information that hints at the nature of the coupling between the two fields. Hence, heterogeneous correlation maps are shown in Fig. 1 and homogeneous correlation maps are shown in Supplementary Material 2.

### North Atlantic and Arctic Oscillations vs. modes of sea ice, NDVI variability, and NCEP/NCAR 40-Year Reanalysis meridional wind & surface air temperatures

Monthly North Atlantic Oscillation – *NAO* – and Arctic Oscillation – *AO* – indices where obtained from the Climate Prediction Center of the National Oceanic & Atmospheric Administration (NOAA, http://www.cpc.ncep.noaa.gov/products/precip/CWlink/daily_ao_index/teleconnections.shtml). Both modes of large-scale atmospheric circulation have been linked to sea ice dynamics in the Arctic^30, 31, 41–43^. Monthly *NAO* indices are based on Rotated Principal Component Analysis (*r*PCA) to monthly mean standardized 500-mb height anomalies using the procedure described by Barnston and Livezey^19^. Monthly *AO* are defined as the leading mode of Empirical Orthogonal Function (EOF) analysis of monthly mean 1000 mb height poleward of 20°N. Monthly *NAO* and *AO* time series for June, July, and August (period 2000–2014) were correlated with the time series for each pixel corresponding to (1) sea ice concentration, (2) *NDVI*, (3) the expansion coefficients of sea ice (***A***), and (4) the expansion coefficients of *NDVI* (***B***) for *EGS*, *JL*, and *LGS*, respectively. Field significance, accounting for the effects of multiplicity in spatially autocorrelated fields, was addressed following the Monte-Carlo-based approach (based on 1,000 iterations) described in Livezey and Chen^44^. Field significance addresses the probability that a given spatial pattern of strong correlations between a given time series and time series in a highly spatially autocorrelated field might be a chance occurrence.

Monthly values for meridional wind – indicating the occurrence or not of southerly warm air advection – and surface air temperature were obtained for the length of the study period 2000–2014 from the NCEP/NCAR Reanalysis project^29^, which provides assimilated data with an horizontal resolution of 2.5° by 2.5° degree, and correlated with monthly indices of the *NAO* and *AO* (Supplementary Material 5).

### Daily meteorological station surface air temperature data

Daily meteorological station surface air temperature data for a set of 12 stations in the Svalbard Archipelago (6 in *W-Sb* and 6 in *E-Sb*; see Supplementary Material 1 for their location and code) were obtained from the Norwegian Meteorological Institute (http://eklima.met.no) for the period 2012–2014 for which daily data was available in all stations. Daily values were averaged into 8-day periods to match the temporal resolution and format of the study (Supplementary Material 6).

## Electronic supplementary material

Supplementary Information
