# Supplementary material for: Disentangling the coupling between sea ice and tundra productivity in Svalbard
Source: Sci Rep. 2017 Aug 17;7:8586. doi: 10.1038/s41598-017-06218-8 (PMC5561272; doi:10.1038/s41598-017-06218-8)

Supplementary Information for

Disentangling the coupling between sea ice and tundra productivity in Svalbard

Marc Macias-Fauria*[1], Stein Rune Karlsen[2] & Bruce C. Forbes[3]

[1]School of Geography & the Environment, University of Oxford, United Kingdom

[2]Norut Northern Research Institute, NO-9294 Tromsø, Norway

[3]Arctic Centre, University of Lapland, FI-96101 Rovaniemi, Finland

* Correspondence to marc.maciasfauria@ouce.ox.ac.uk

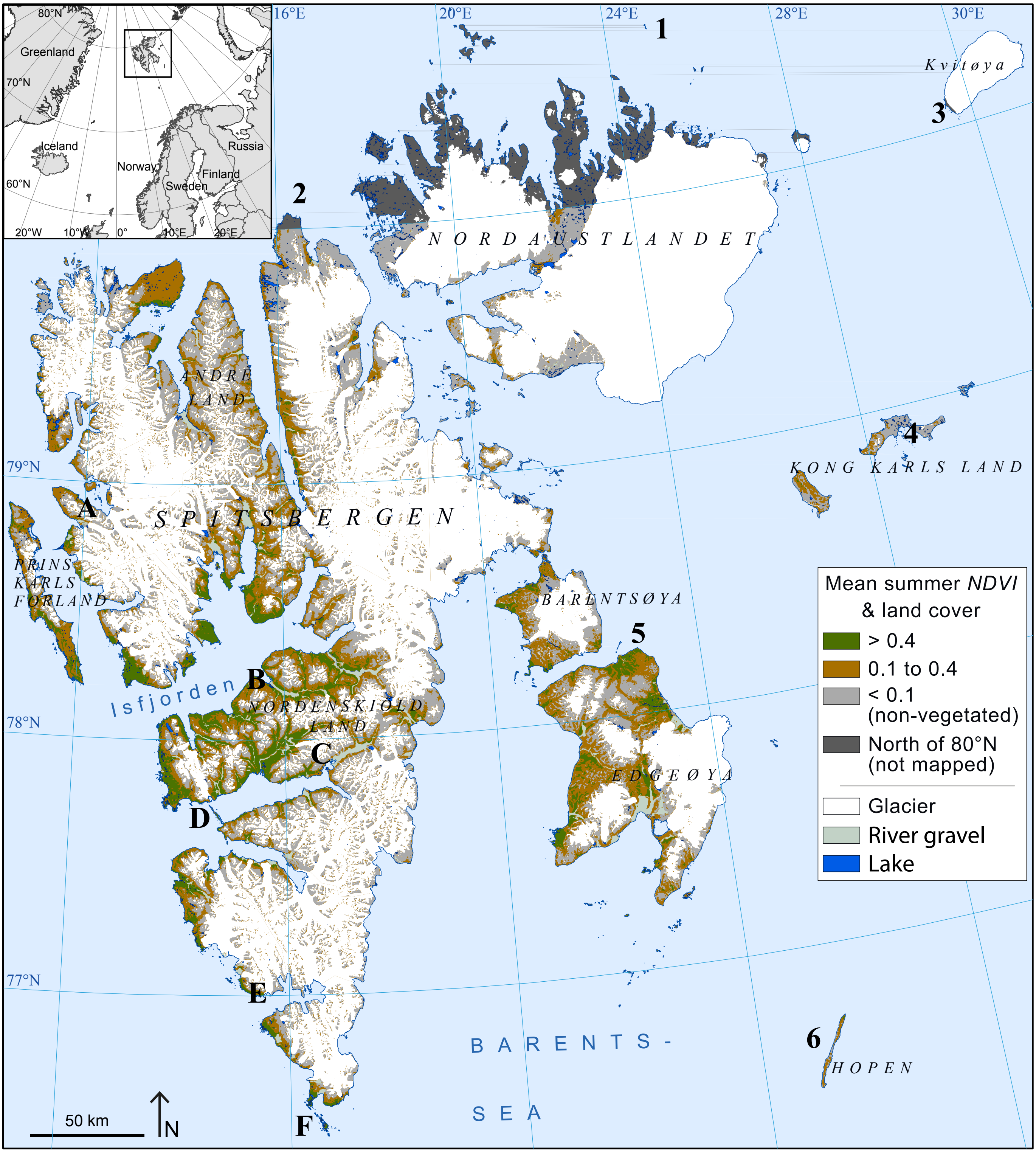

Supplementary Material 1. Study area of Svalbard Archipelago showing cloud-corrected MODIS-NDVI values for the period 4th July to 3rd August ("*summer NDVI*"). The values are the arithmetic mean for the 2000–2014 period. NDVI values > 0.2 indicate tundra vegetation cover, and <0.2, very sparse or non-vegetated areas. The main geographical names used in the main text are mapped. For a detailed description of vegetation cover in Svalbard refer to Johansen et al. (2012)[21]. Capital letters refer to meteorological stations located in *W-Sb* (see ***Supplementary Material 5***): **A**: Ny Ålesund (station code 99910); **B**: Longyearbyen airport (station code 99840); **C**: Sveagruva (station code 99760); **D**: Akseløya (station code 99765); **E**: Hornsund (station code 99754); **F**: Sørkappøya (station code 99752). Numbers refer to meteorological stations located in *E-Sb* (see ***Supplementary Material 5***): **1**: Karl XII-Øya (station code 99935); **2**: Verlegenhuken (station code 99927); **3**: Kvitøya (station code 99938); **4**: Kongsøya (station code 99740); **5**: Edgeøya (station code 99735); **6**: Hopen (station code 99720). The map was generated using ArcMap (version 10.3; https://www.esri.com) with the ArcPy Python site package, used herein under license.

Supplementary Material 2
Statistics of the Singular Vector Decomposition

See ***Methods*** for details.

1. Statistics table for the first 3 *SVs* in each *SVD* computed. Only the 1st *SV* in each *SVD* was used in the present study (*highlighted in orange*).

			SCF_k	NCF_k	ρ_k
E-Sb	*GSE*	1st SV	62.63%	19.47%	0.66
		2nd SV	27.82%	12.98%	0.81
		3rd SV	4.99%	5.49%	0.78
		Total	95.45%	37.95%	-
	JL	1st SV	80.87%	23.20%	0.62
		2nd SV	12.94%	9.28%	0.60
		3rd SV	2.00%	3.65%	0.82
		Total	95.81%	36.13%	-
	GSL	1st SV	70.84%	17.86%	0.58
		2nd SV	15.43%	8.33%	0.77
		3rd SV	4.85%	4.67%	0.55
		Total	91.11%	30.87%	-
W-Sb	*GSE*	1st SV	84.19%	31.27%	0.80
		2nd SV	6.51%	8.70%	0.78
		3rd SV	3.56%	6.43%	0.78
		Total	94.27%	46.41%	-
	JL	1st SV	89.38%	29.23%	0.74
		2nd SV	4.50%	6.56%	0.71
		3rd SV	1.28%	3.49%	0.83
		Total	95.15%	39.28%	-
	GSL	1st SV	80.40%	23.37%	0.65
		2nd SV	10.04%	8.26%	0.72
		3rd SV	2.76%	4.33%	0.82
		Total	93.20%	35.96%	-

SCF_k: squared covariance fraction for each mode *k*.
NCF_k: normalised root-mean squared covariance fraction for each mode *k*.
ρ_k: correlation between the expansion coefficients for each mode *k*.

2. Singular Vector Maps – For the 1st *SV* only. Note that the sign of the vector is arbitrary. Maps were generated using Matlab (version R2016b; https://www.mathworks.com), used herein under license.

Singular Vector Maps – *E-Sb, GSE*

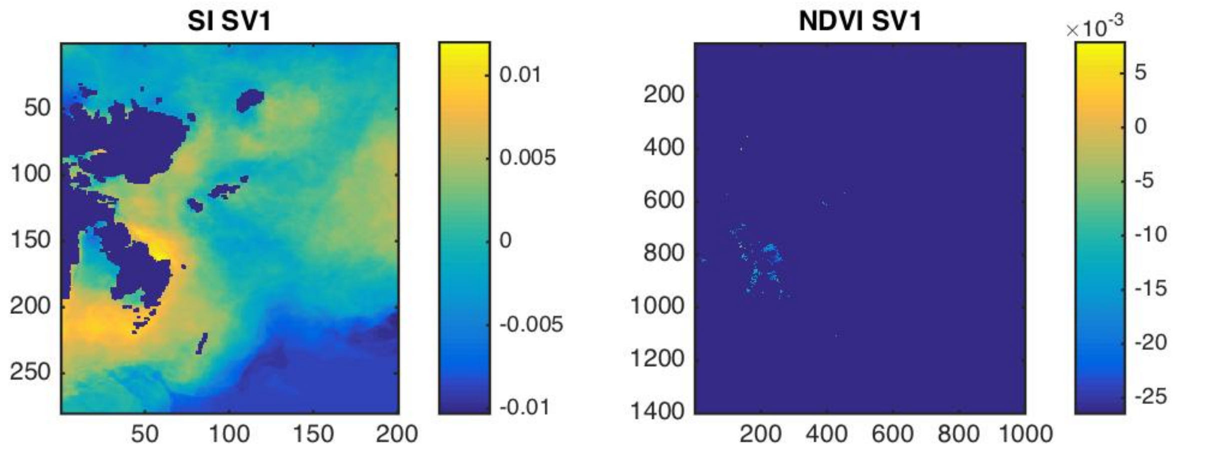

Singular Vector Maps – *E-Sb, JL*

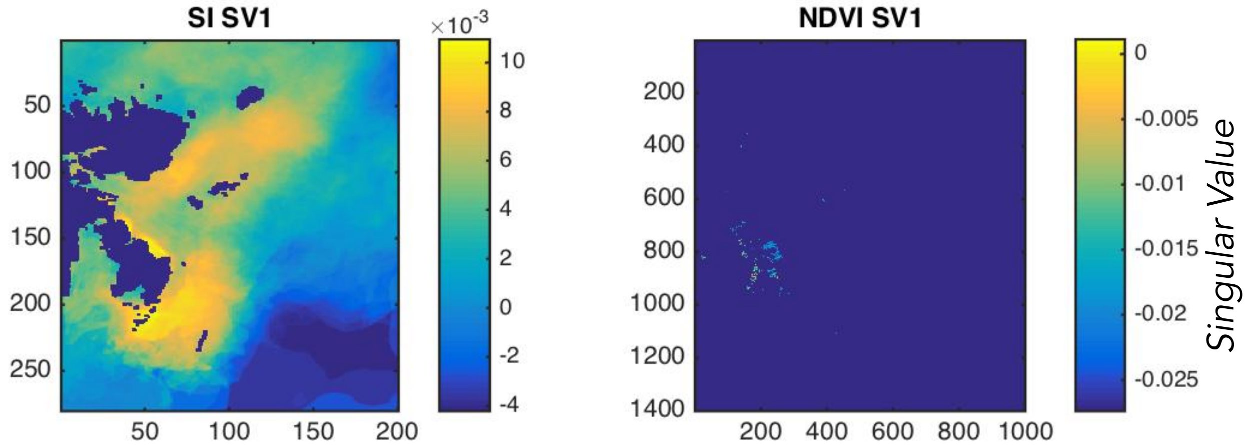

Singular Vector Maps – *E-Sb, GSL*

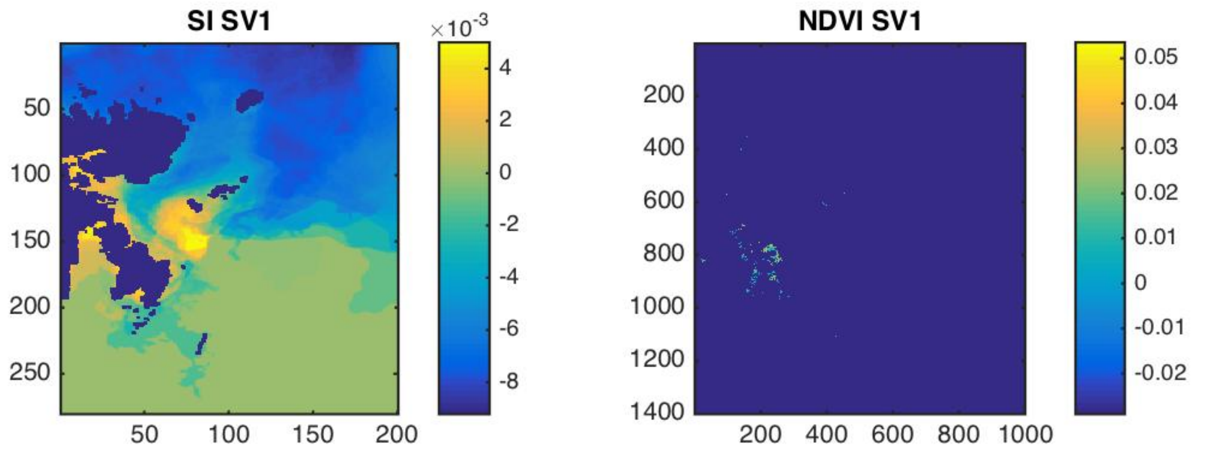

Singular Vector Maps – For the 1st SV only

Singular Vector Maps – *W-Sb, GSE*

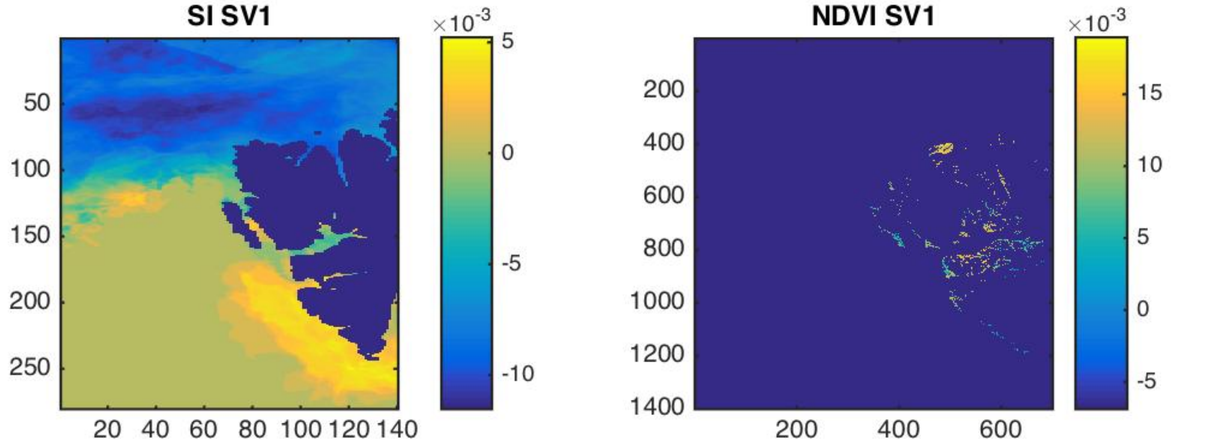

Singular Vector Maps – *W-Sb, JL*

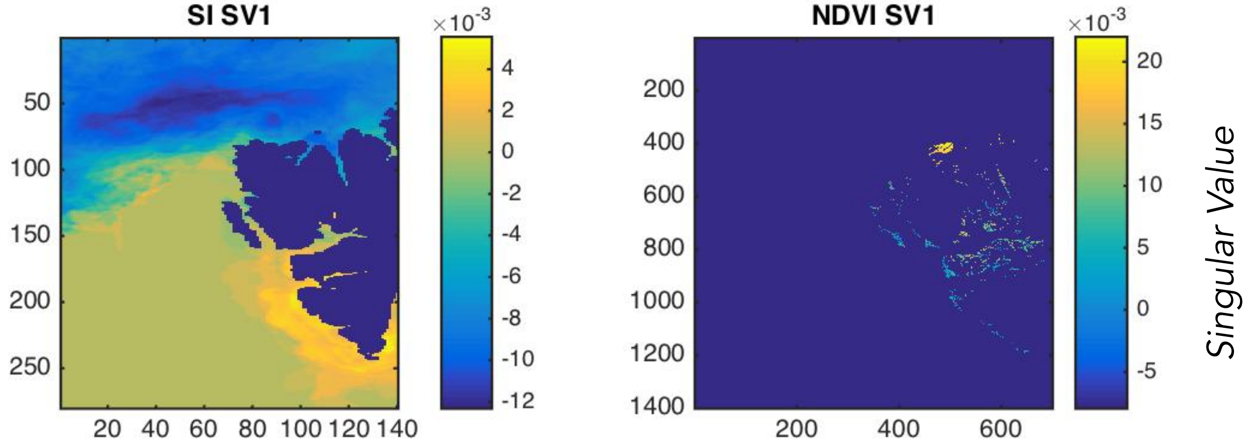

Singular Vector Maps – *W-Sb, GSL*

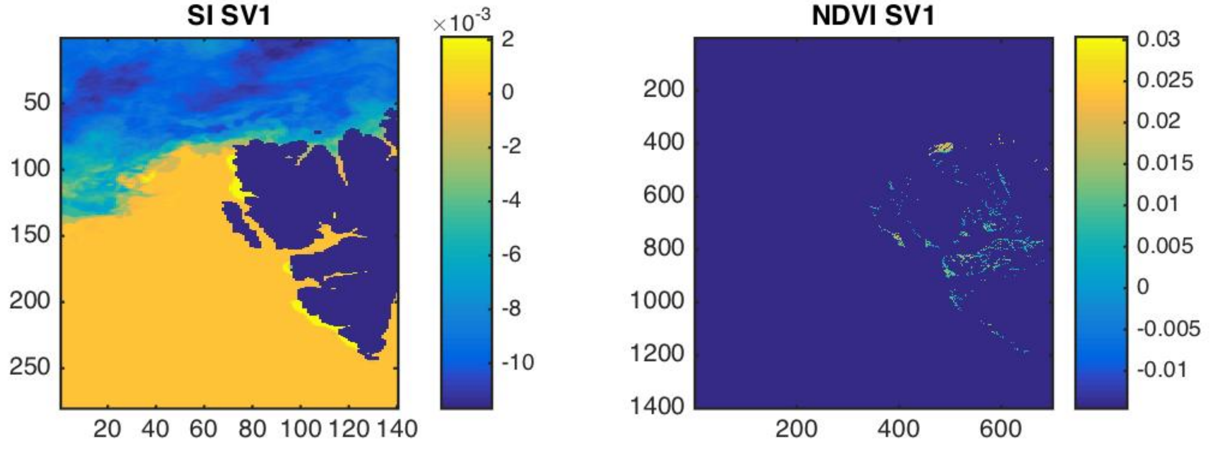

3. Expansion Coefficient Time Series – For the 1st *SV* only. Note that the sign of the coefficient is arbitrary.

Expansion Coefficient Time Series – *E-Sb*, *GSE*

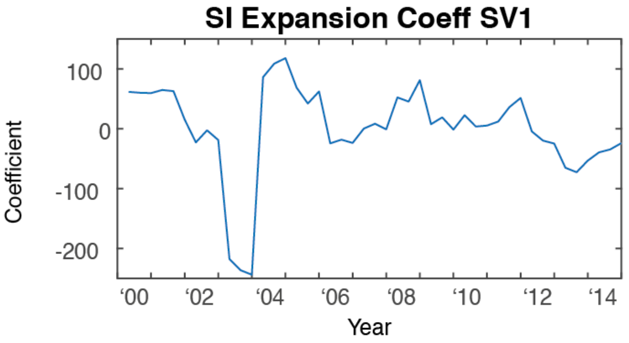

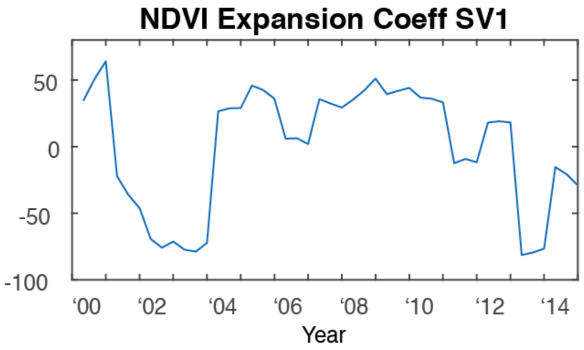

Expansion Coefficient Time Series – *E-Sb*, *JL*

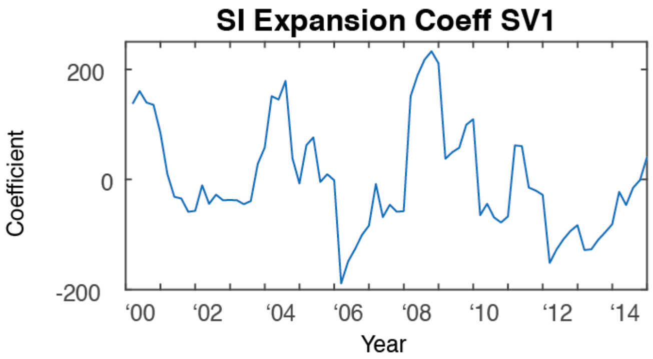

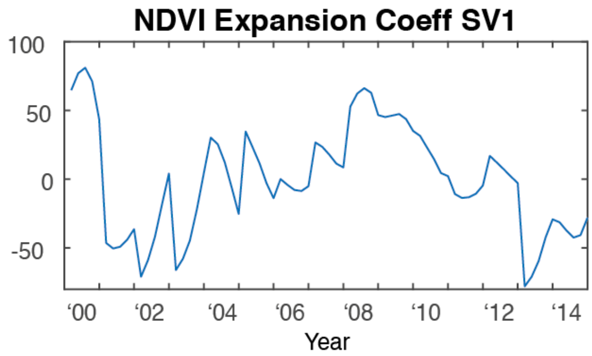

Expansion Coefficient Time Series – *E-Sb*, *GSL*

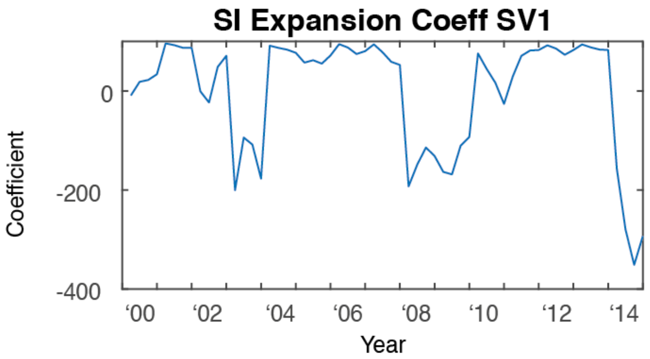

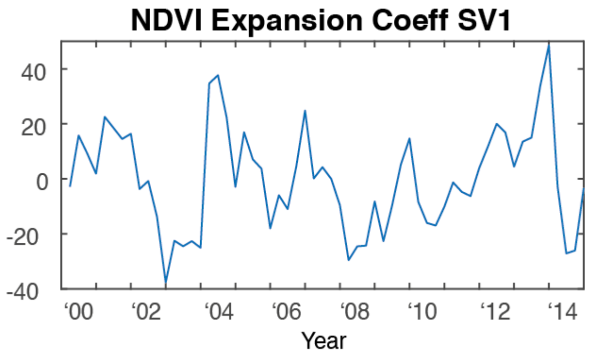

Expansion Coefficient Time Series – For the 1st SV only

Expansion Coefficient Time Series – *W-Sb, GSE*

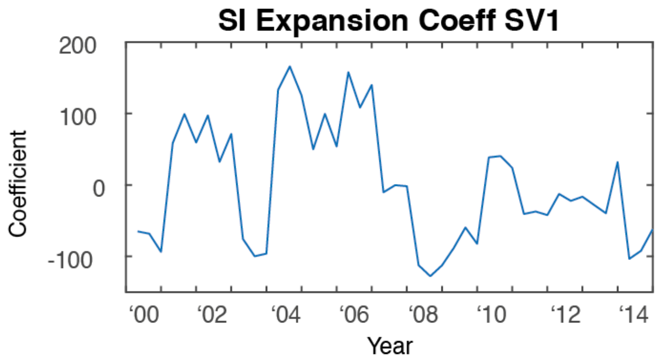

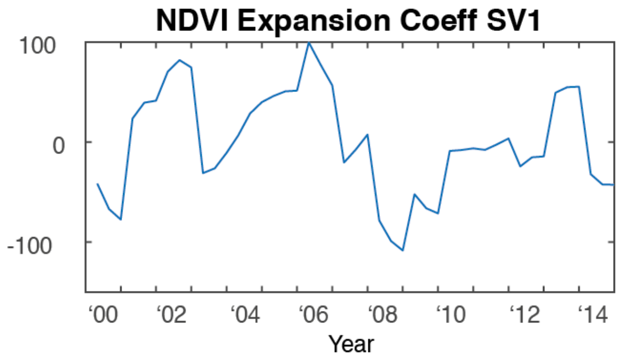

Expansion Coefficient Time Series – *W-Sb, JL*

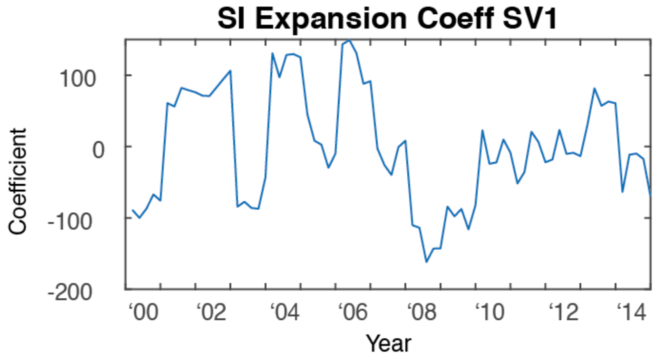

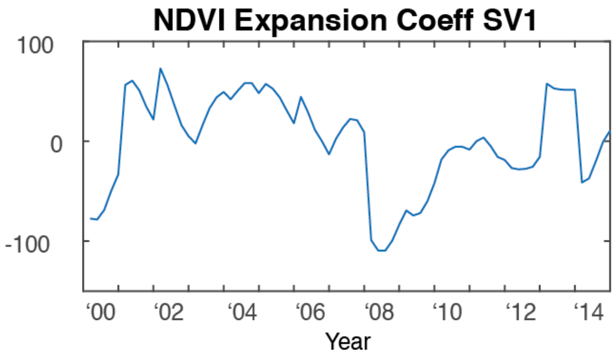

Expansion Coefficient Time Series – *W-Sb, GSL*

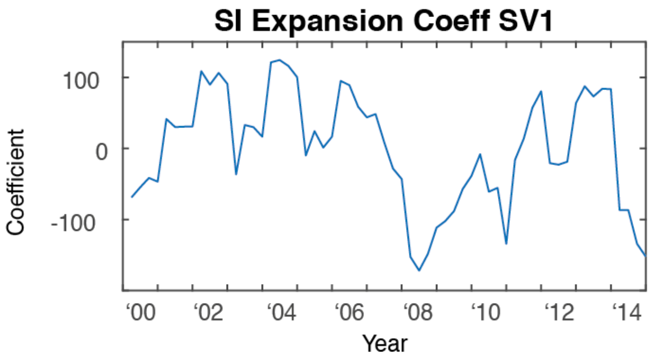

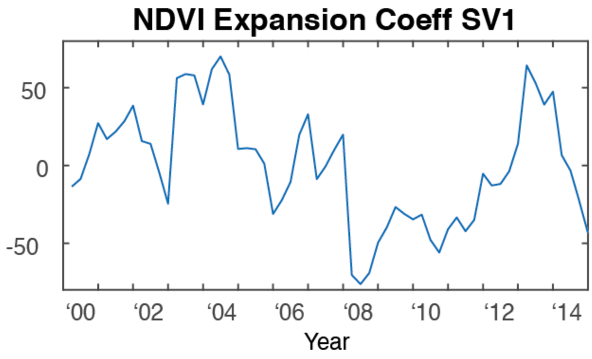

4. Homogeneous Correlations – For the 1st *SV* only. Note that the sign of the correlation is arbitrary due to the arbitrary sign of the *SV*. Maps were generated using Matlab (version R2016b; https://www.mathworks.com), used herein under license.

Homogeneous Correlations – *E-Sb*, *GSE*

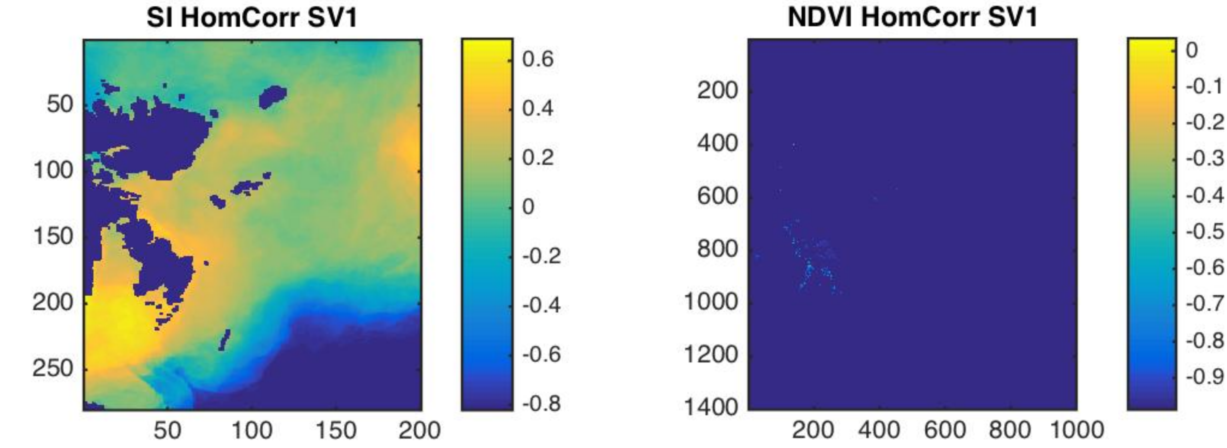

Homogeneous Correlations – *E-Sb*, *JL*

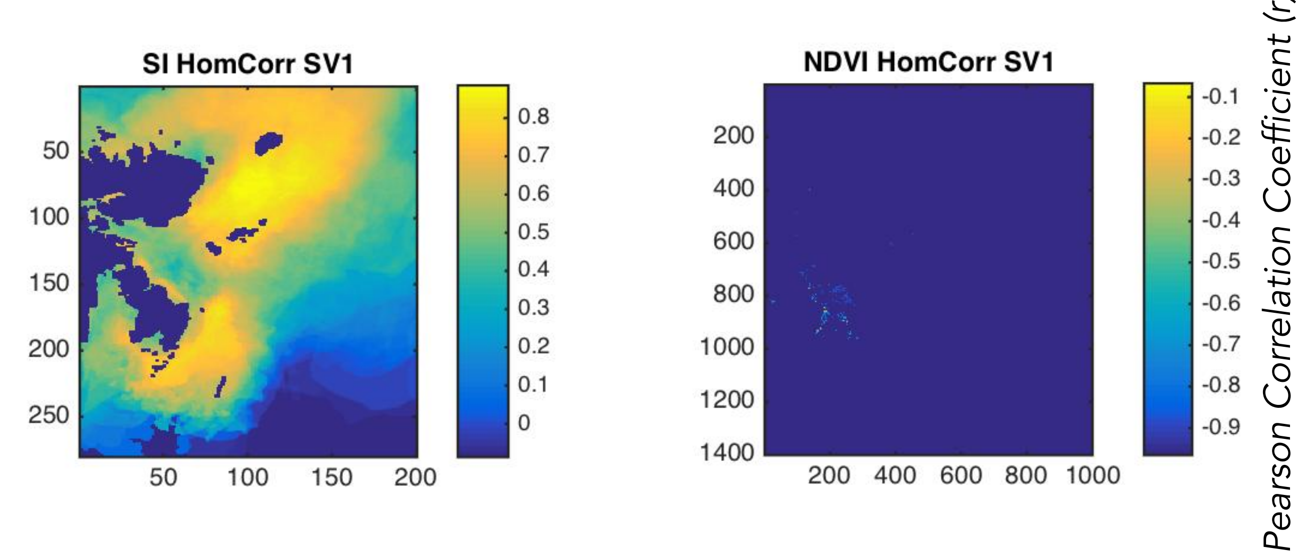

Homogeneous Correlations – *E-Sb*, *GSL*

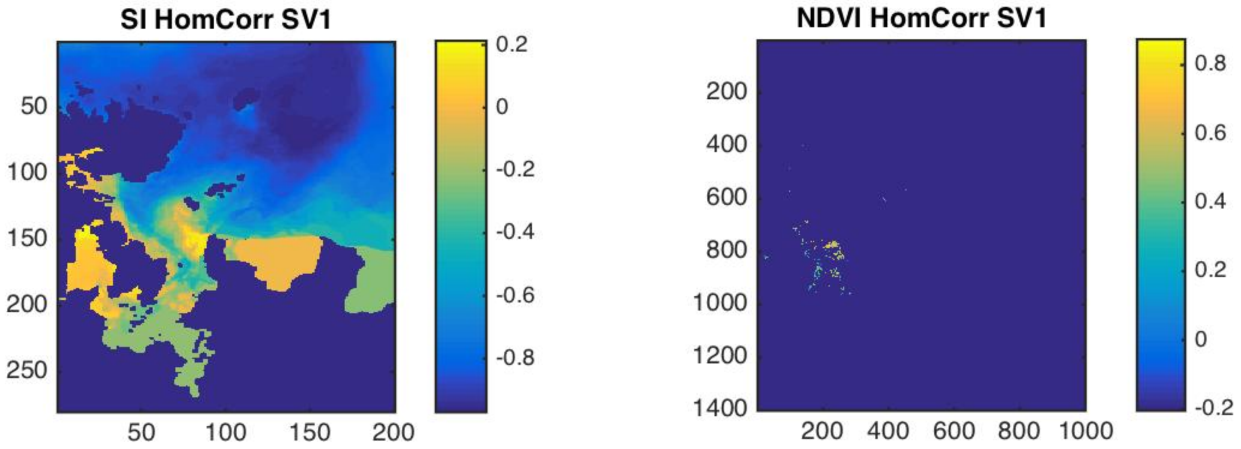

Homogeneous Correlations – For the 1st SV only

Homogeneous Correlations – *W-Sb, GSE*

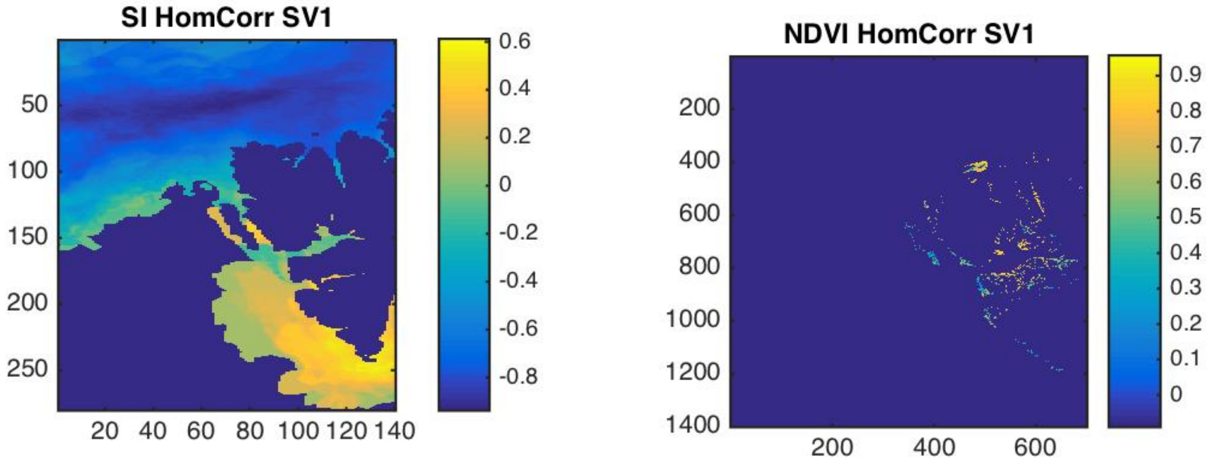

Homogeneous Correlations – *W-Sb, JL*

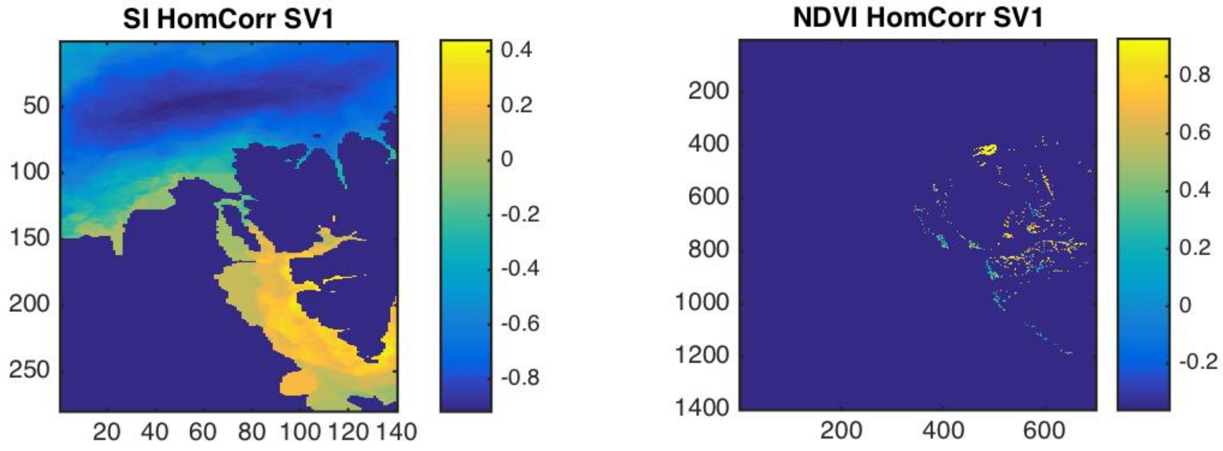

Pearson Correlation Coefficient (r)

Homogeneous Correlations – *W-Sb, GSL*

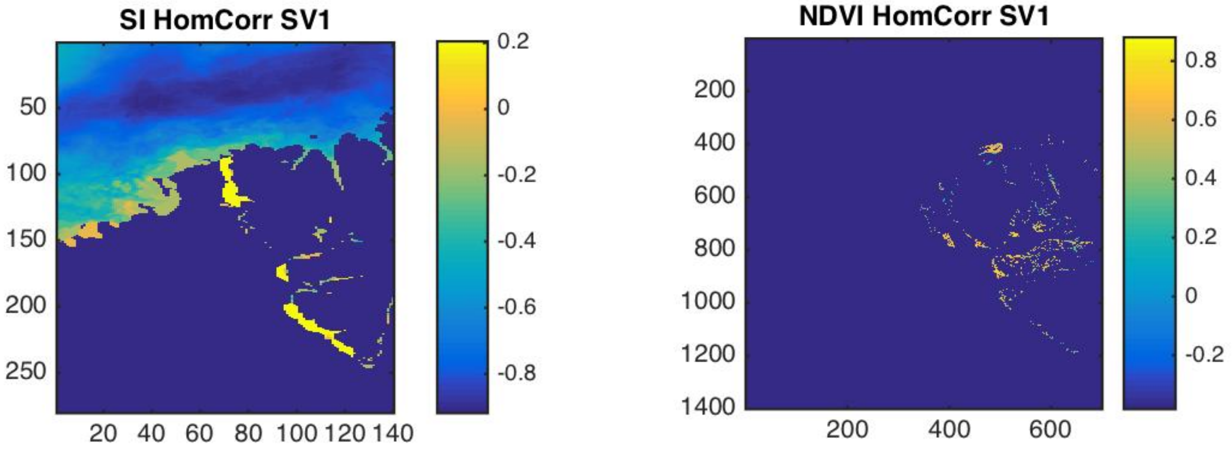

Supplementary Material 3.

Eight-day mean sea ice concentration (From April 23 to September 30; period 2000-2014). See *Methods* for details. Maps were generated using Matlab (version R2016b; https://www.mathworks.com), used herein under license.

Eight-day Period 1 – April 23rd- 30th

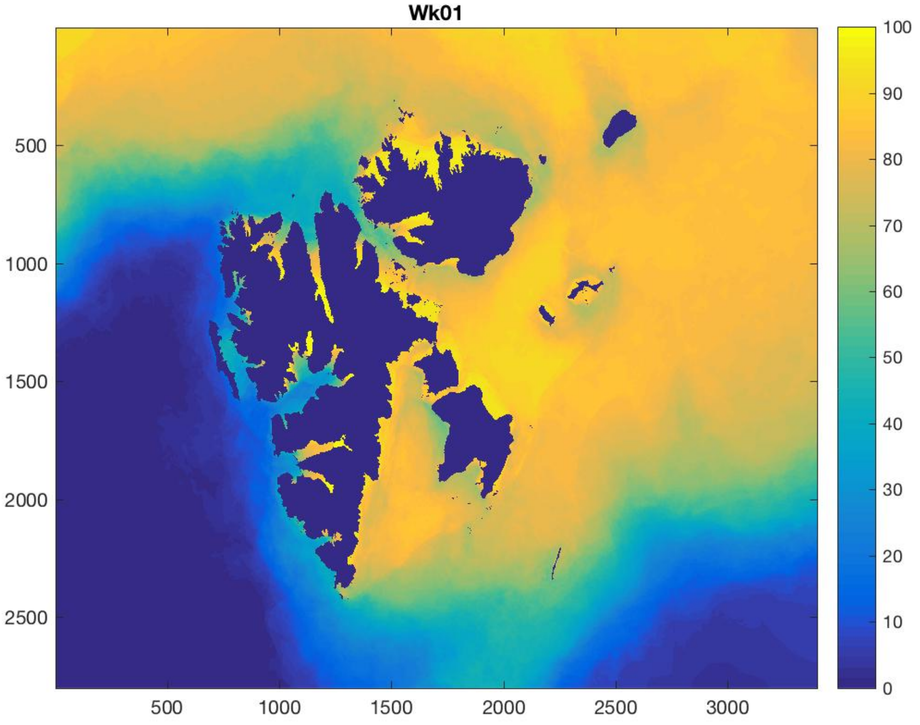

Eight-day Period 2 – May 1st- 7th

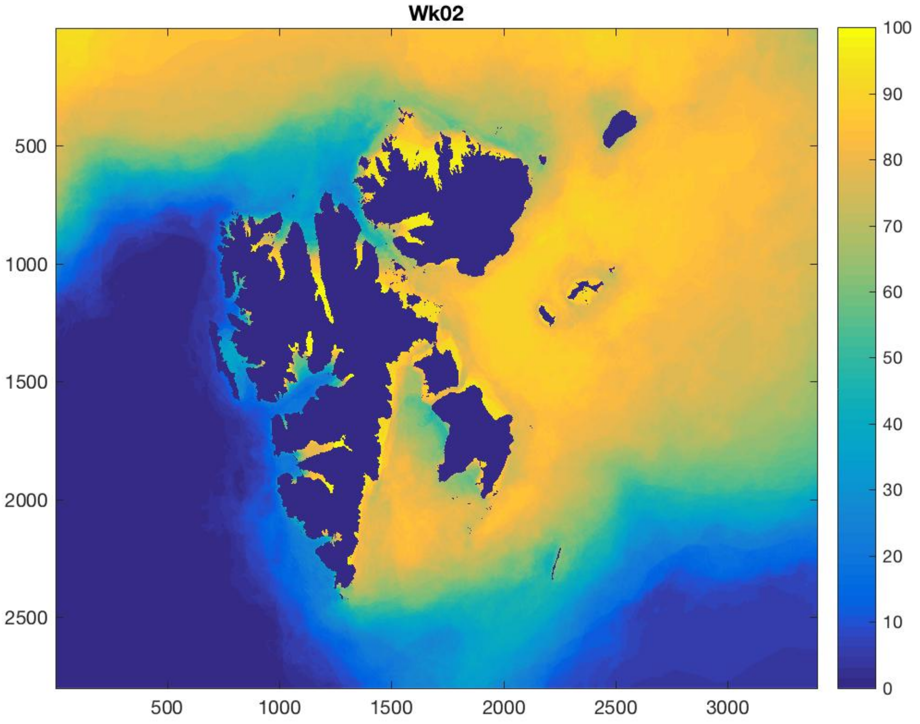

Eight-day Period 3 – May 8th- 15th

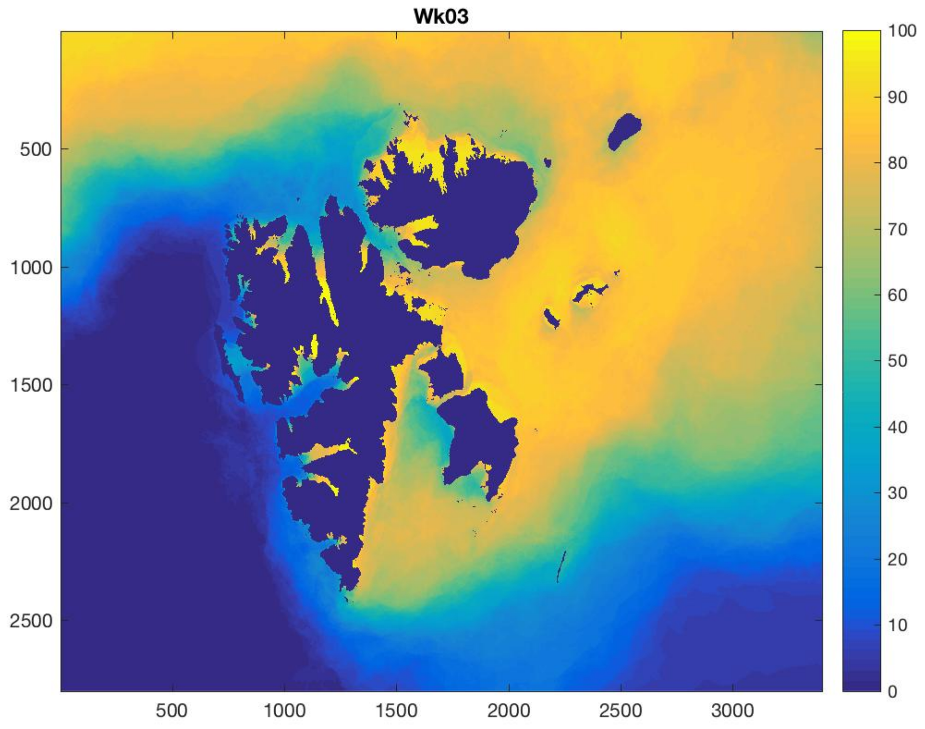

Eight-day Period 4 – May 16th- 23th

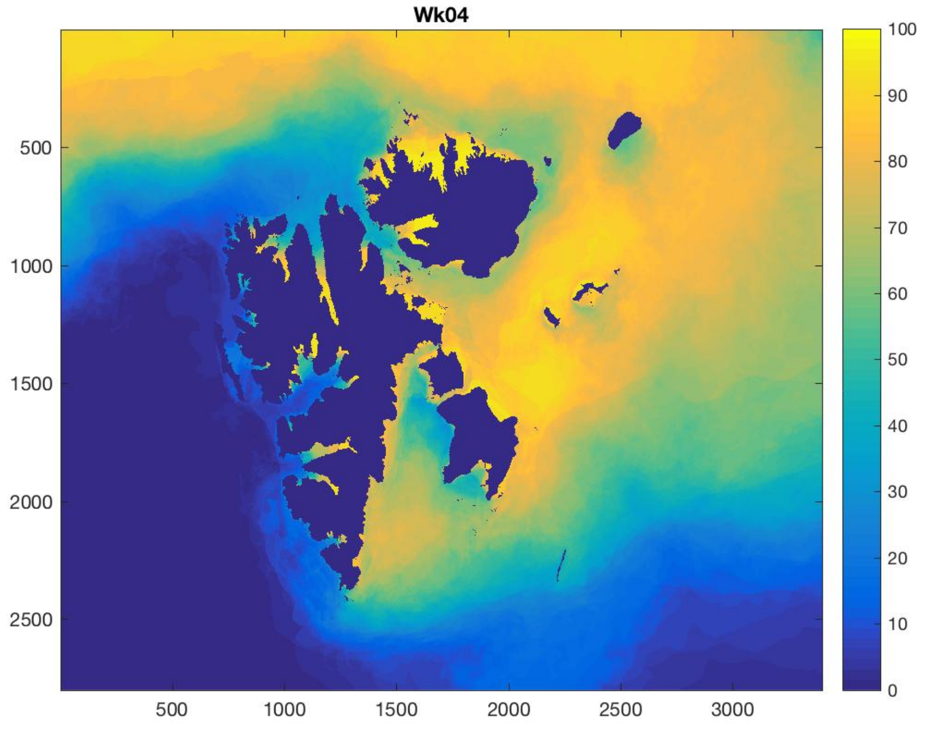

Eight-day Period 5 – May 24th- June 1st

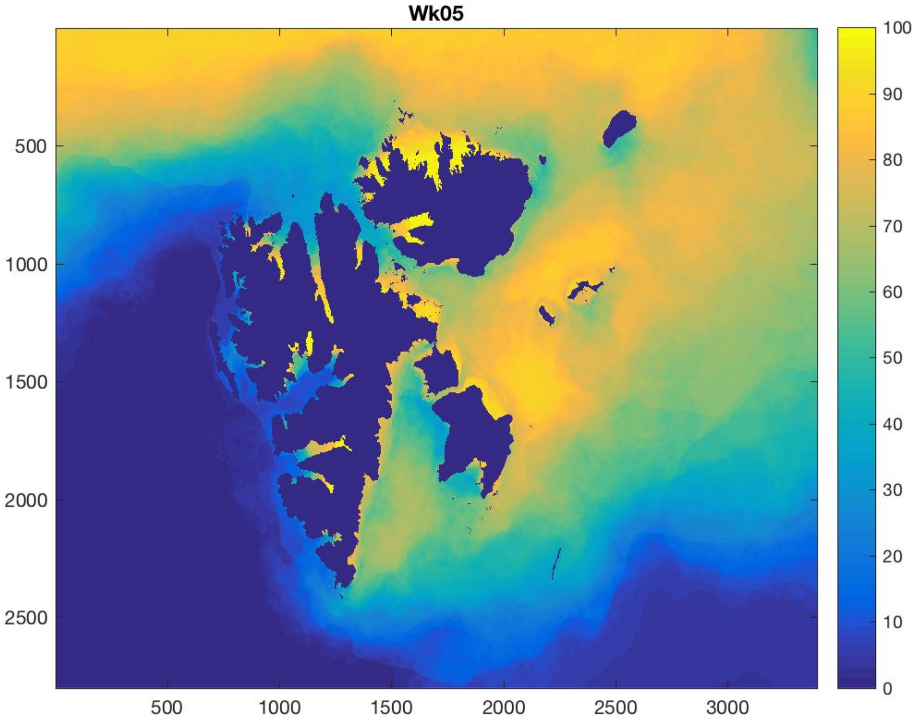

Eight-day Period 6 – June 2nd-June 10th

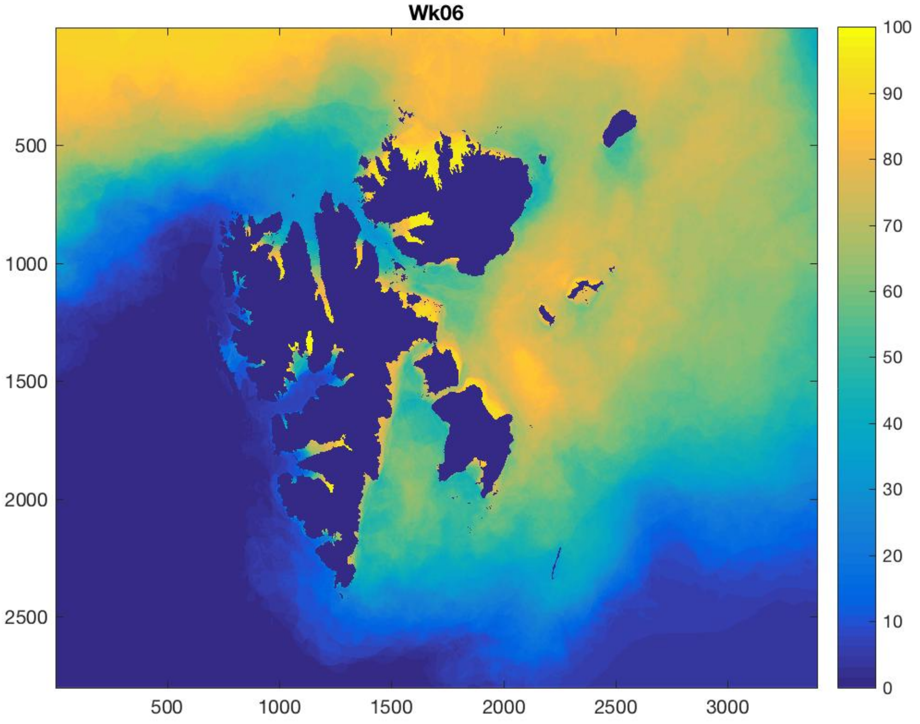

Eight-day Period 7 – June 10th- 17th – GSE, GS

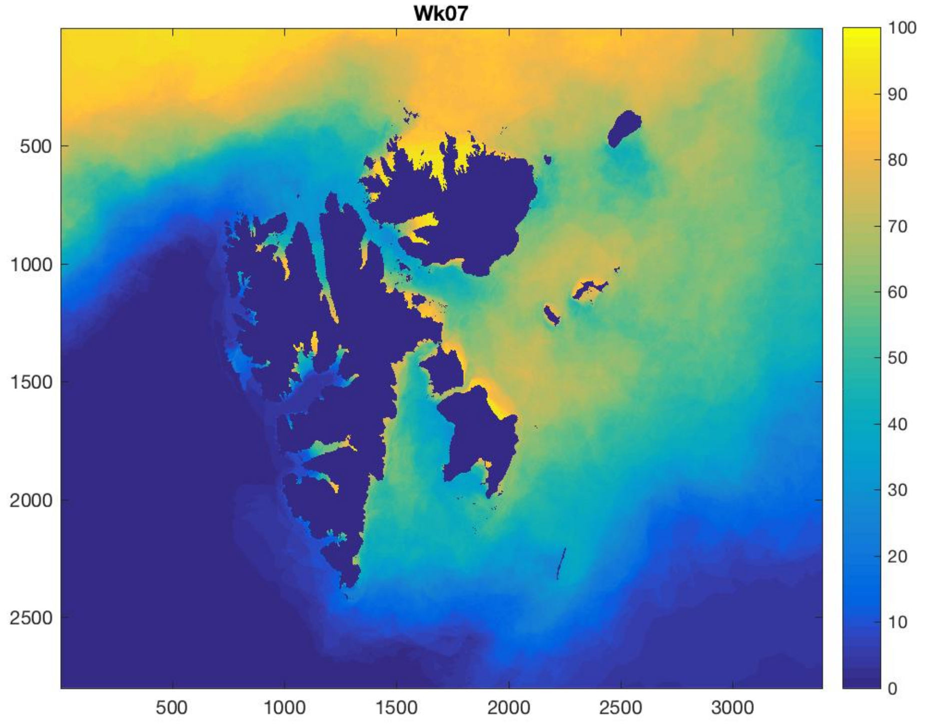

Eight-day Period 8 – June 18th- 25th – GSE, GS

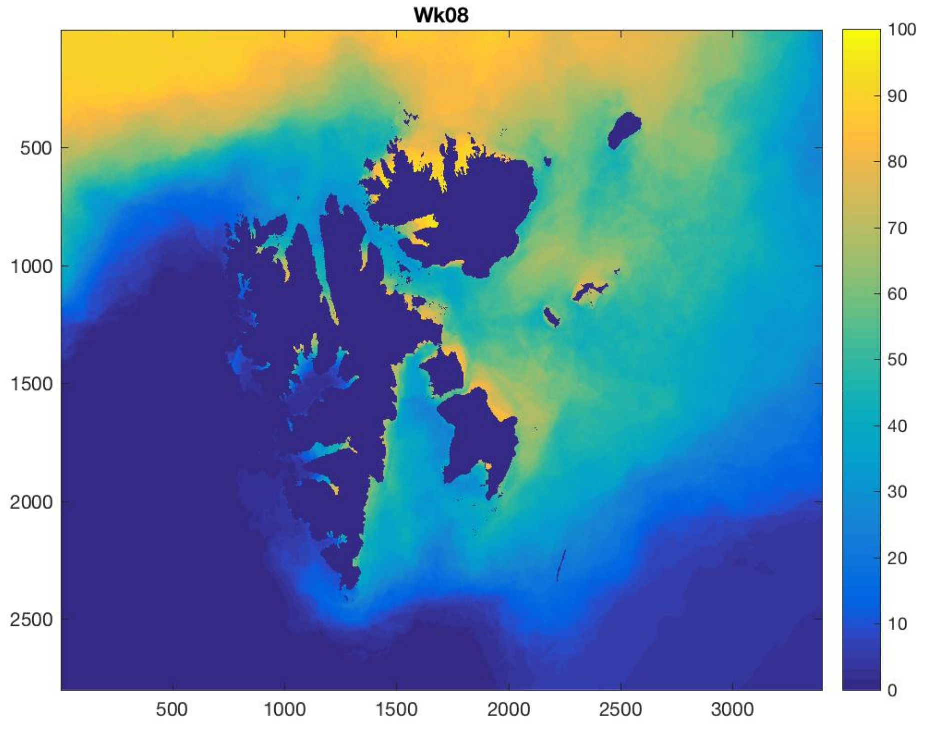

Eight-day Period 9 – June 26th- July 3rd – GSE, GS, JL

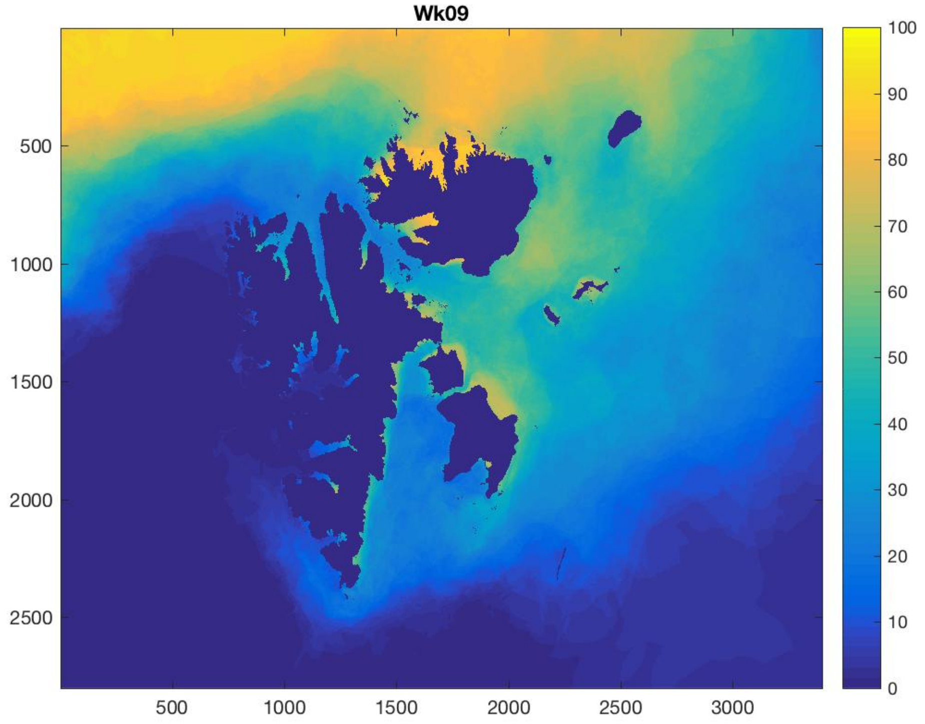

Eight-day Period10 – July 4th- July 11th – GS, JL

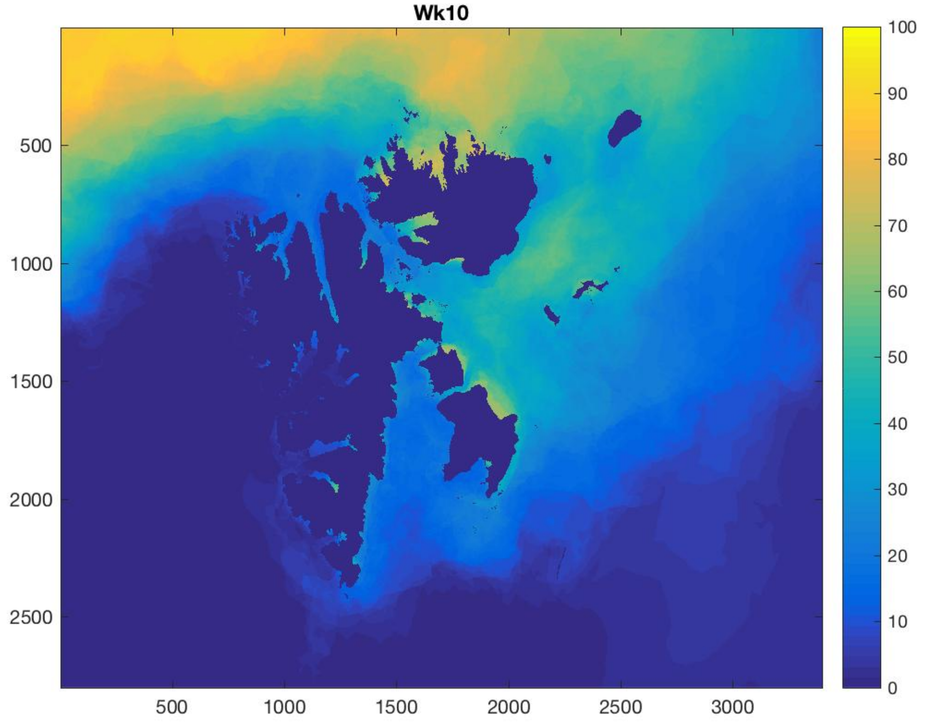

Eight-day Period 11 – July 12th- July 19th – GS, JL

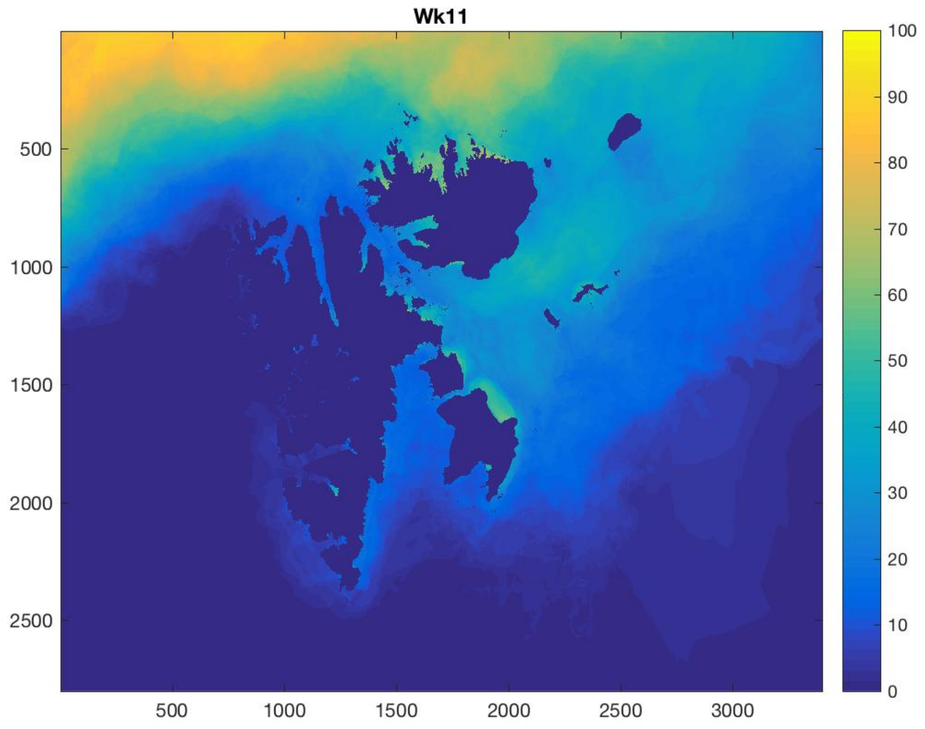

Eight-day Period 12 – July 20th- July 27th – GS, JL

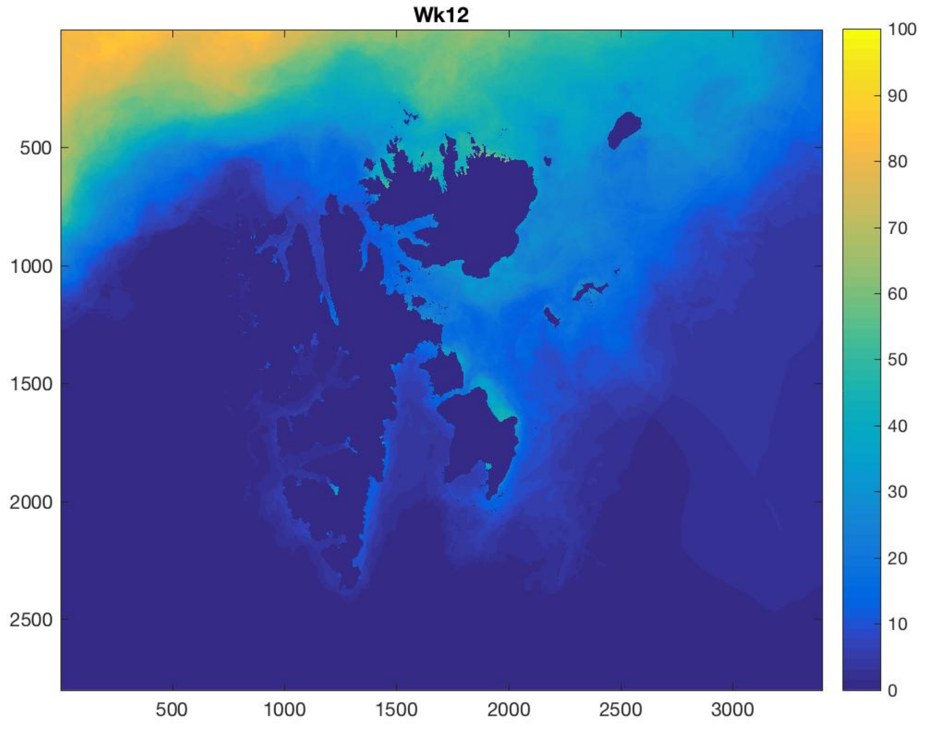

Eight-day Period 13 – July 28th- August 4th – GS, JL

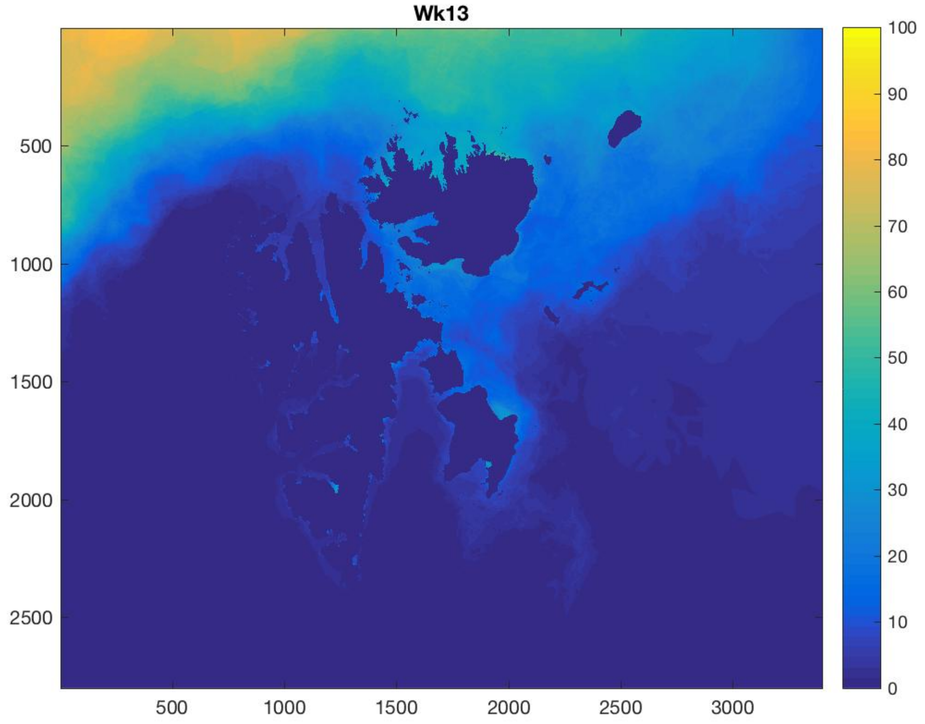

Eight-day Period 14 – August 5th- August 13th – GSL, GS

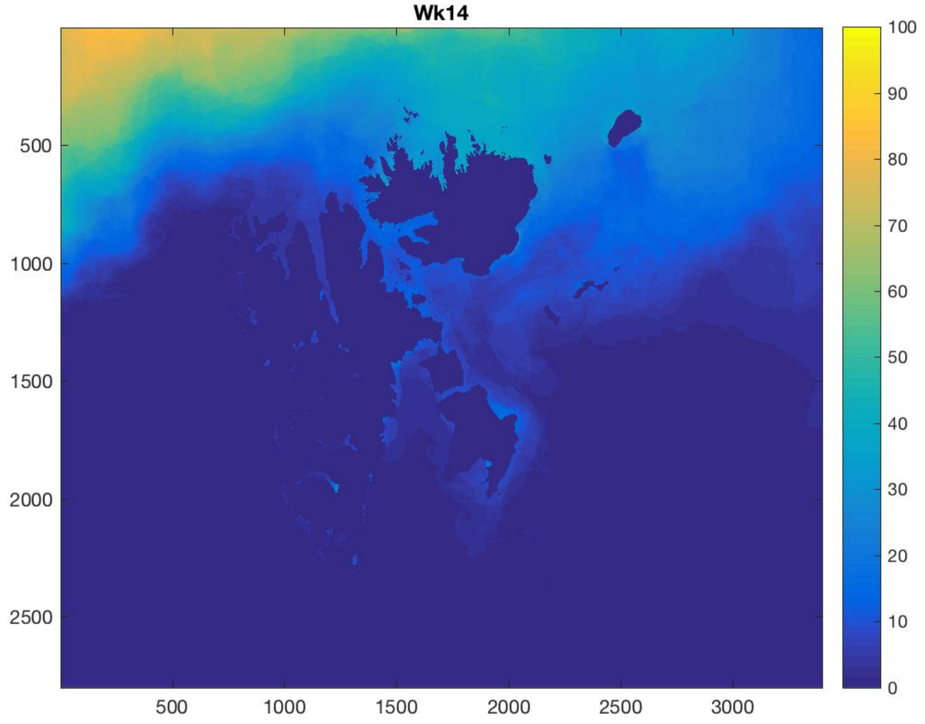

Eight-day Period 15 – August 14th- August 20th – GSL, GS

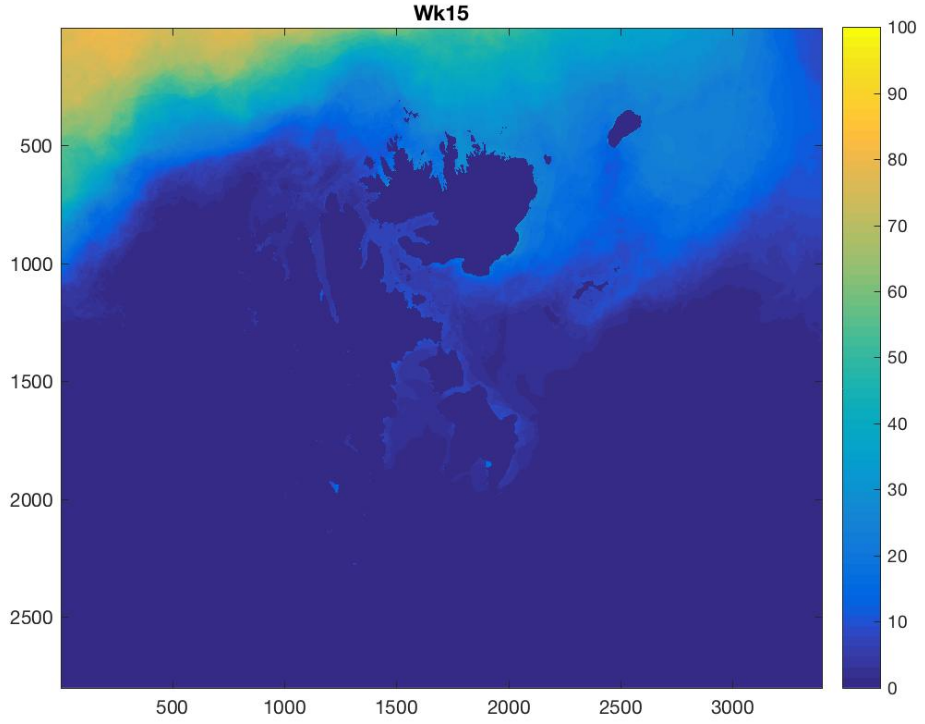

Eight-day Period 16 – August 21st- August 27th – GSL, GS

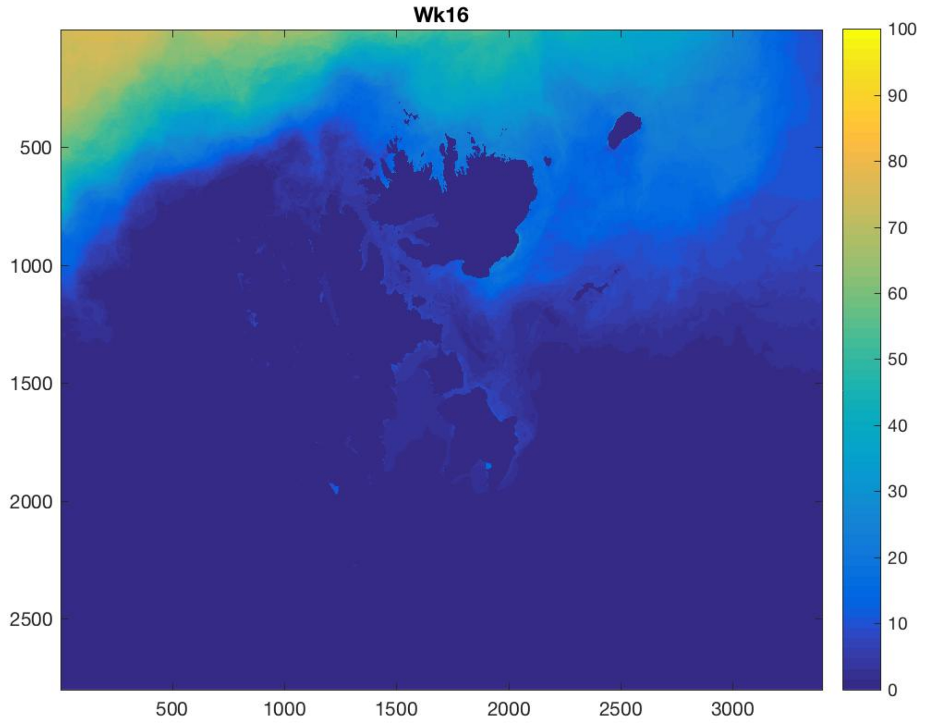

Eight-day Period 17 – August 28st- September 5th – GSL, GS

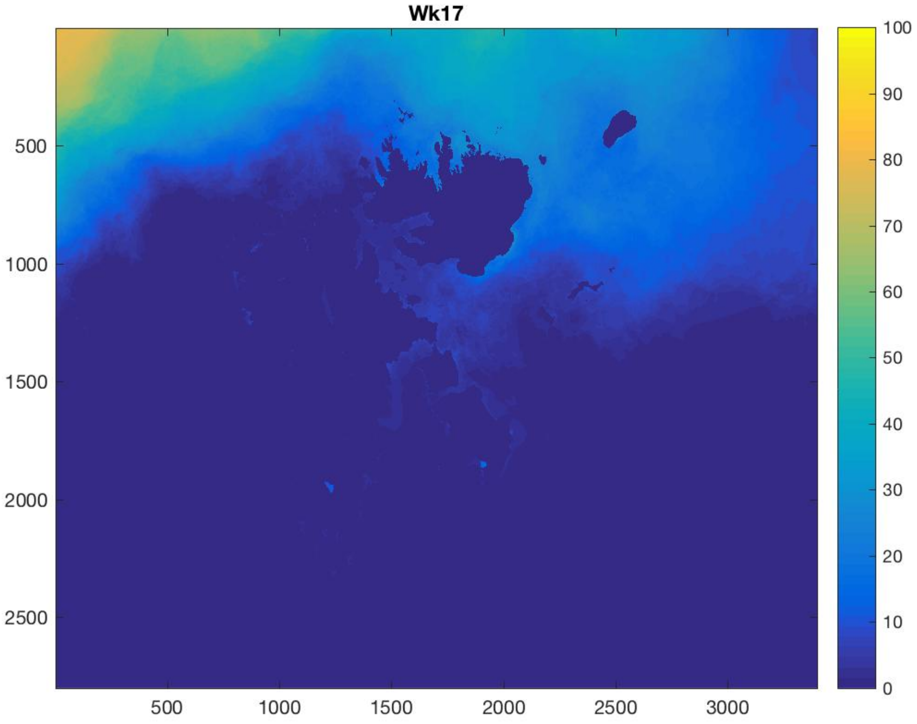

Eight-day Period 18 – September 6th - September 13th

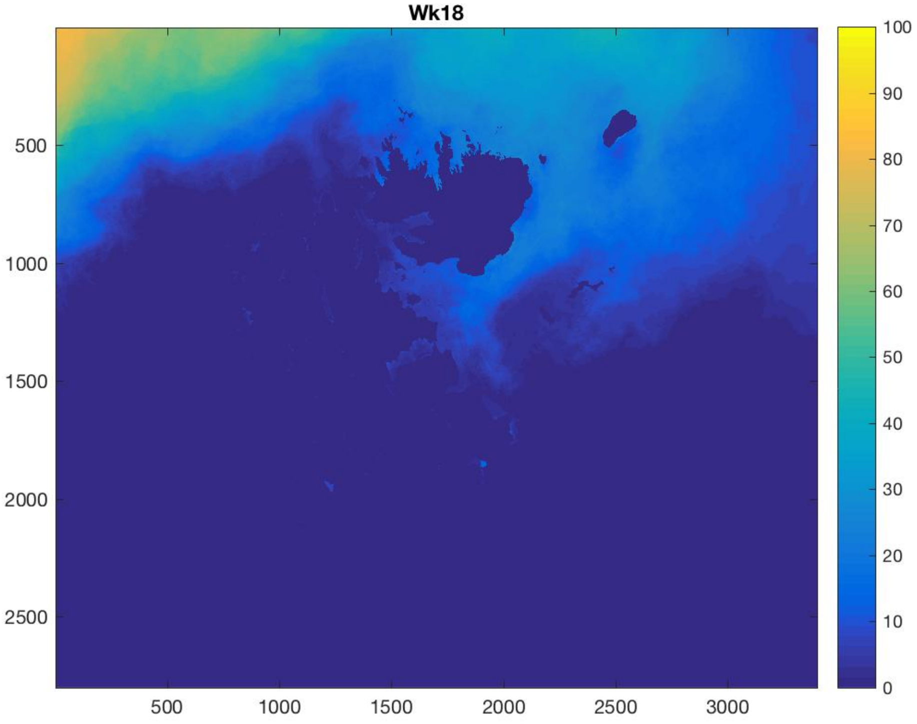

Eight-day Period 19 – September 14th - September 21st

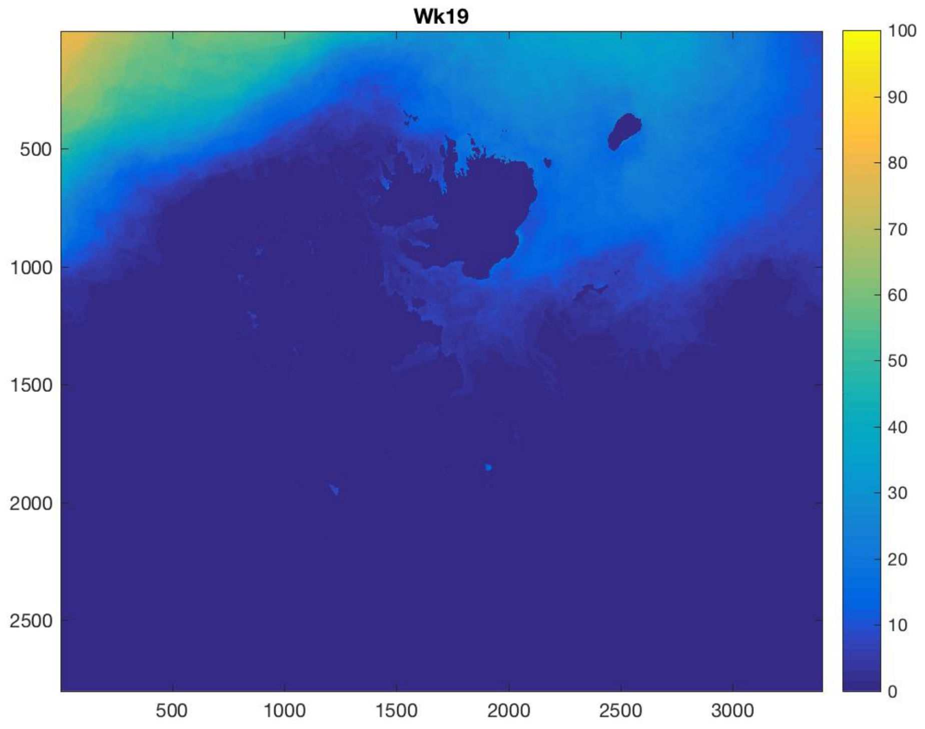

Eight-day Period 20 – September 22nd- September 30th

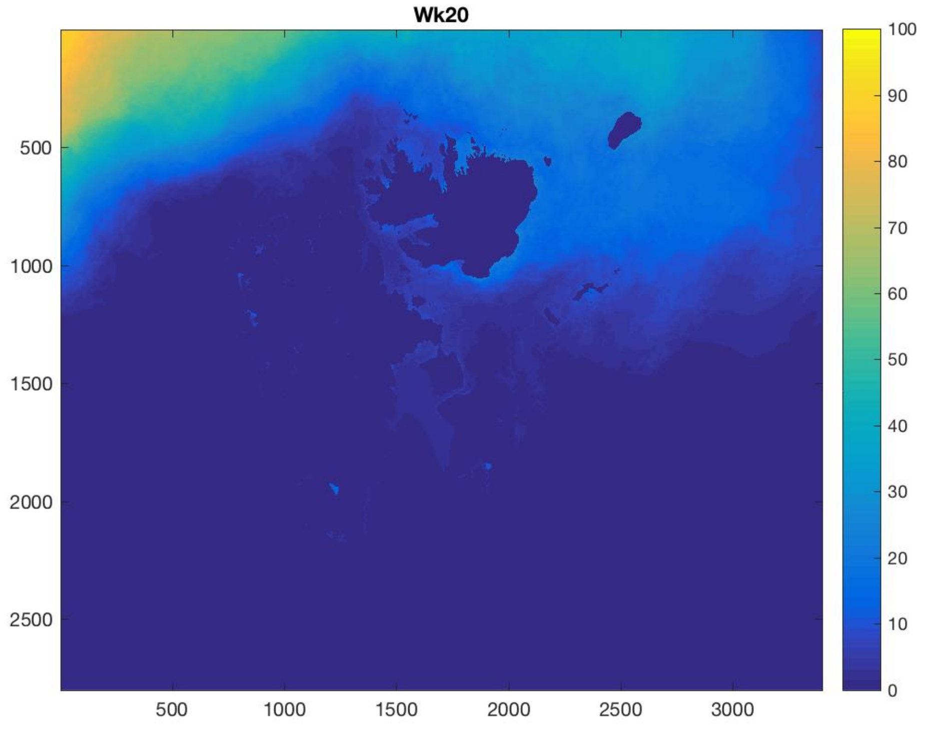

Supplementary Material 4

North Atlantic (*NAO*) and Arctic Oscillation (*AO*) *vs.* Sea Ice concentration and *NDVI* in the Svalbard Archipelago.

See Methods.

FIELD SIGNIFICANCE (see Methods)

**	$p<0.01$
*	$p<0.05$
N.S.	*Non-significance*

Sea Ice EGS *vs.* NAO June

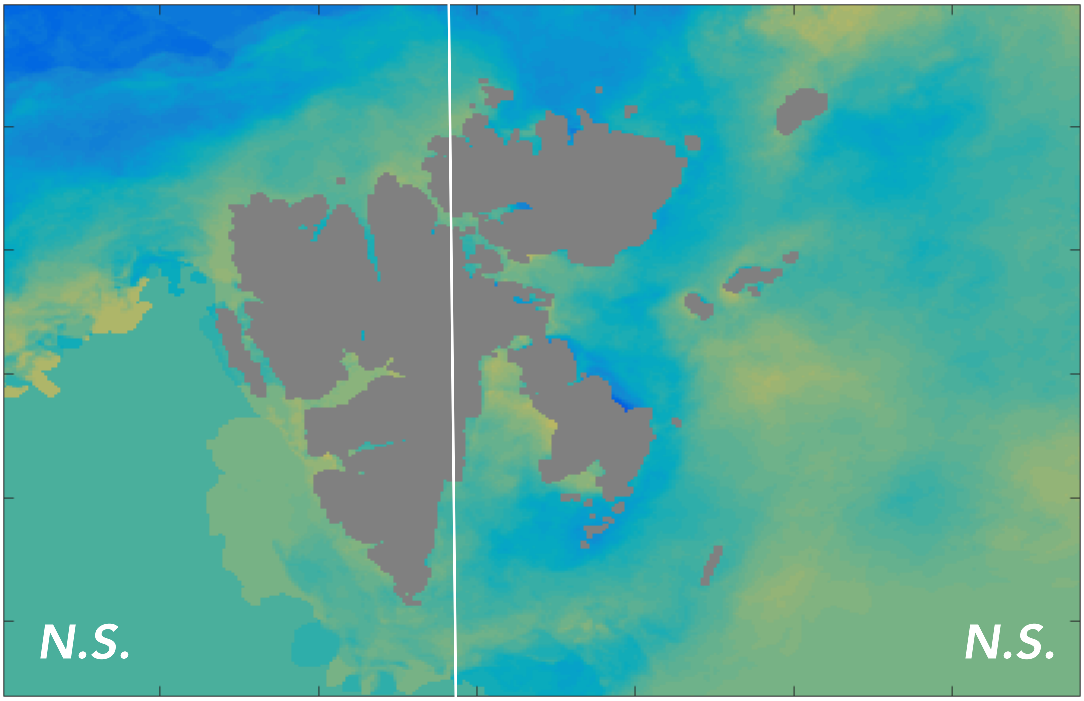

Sea Ice JL *vs.* NAO July

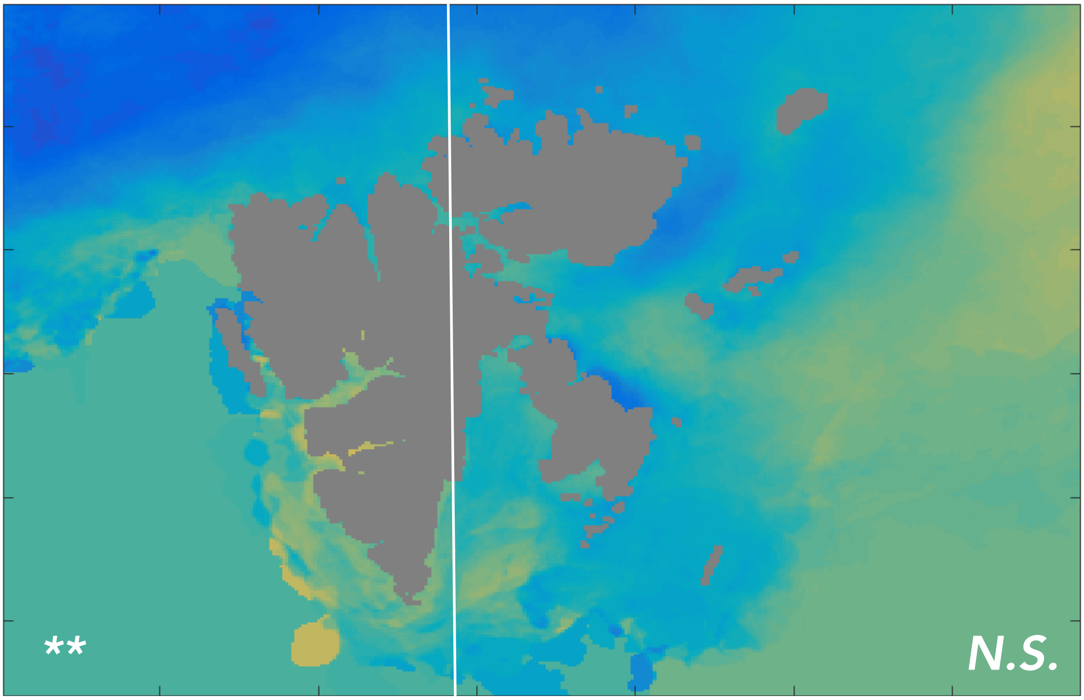

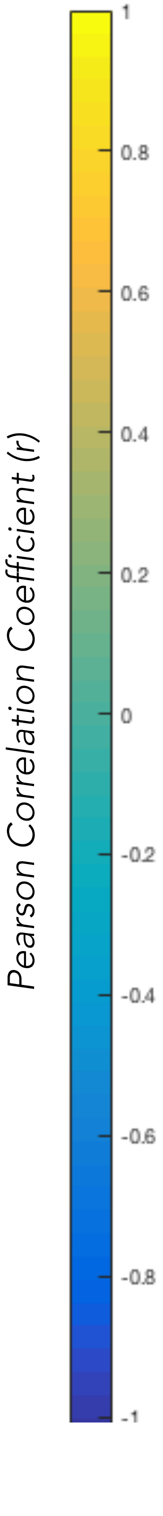

All maps were generated using Matlab (version R2016b; https://www.mathworks.com) and ArcMap (version 10.2; https://www.esri.com), used herein under license.

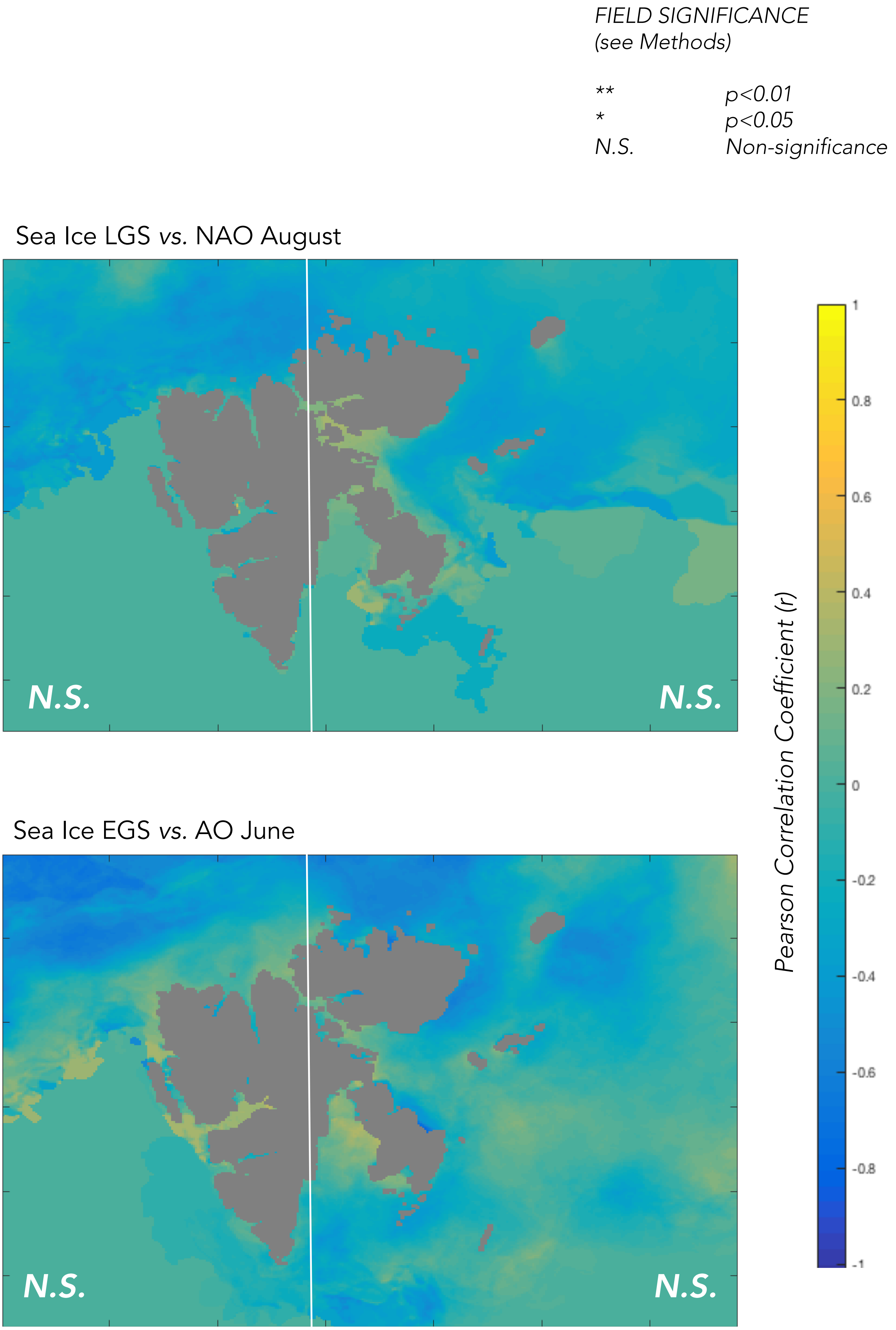
FIELD SIGNIFICANCE
(see Methods)
** p<0.01
* p<0.05
N.S. Non-significance
Sea Ice LGS vs. NAO August
N.S.
N.S.
Sea Ice EGS vs. AO June
N.S.
N.S.
Pearson Correlation Coefficient (r)
1
0.8
0.6
0.4
0.2
0
-0.2
-0.4
-0.6
-0.8
-1

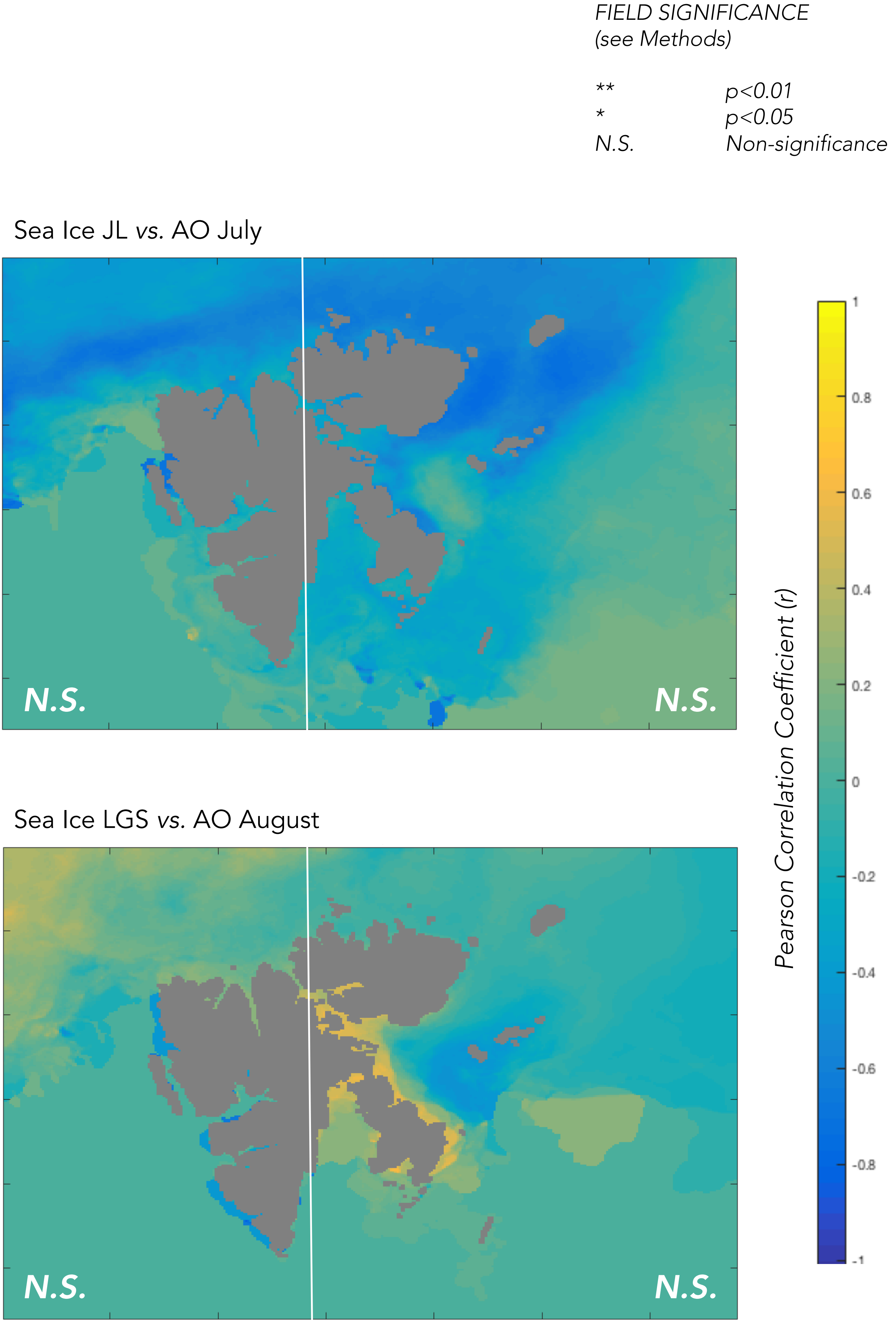
FIELD SIGNIFICANCE
(see Methods)
** p<0.01
* p<0.05
N.S. Non-significance
Sea Ice JL vs. AO July
N.S.
N.S.
Sea Ice LGS vs. AO August
N.S.
N.S.
Pearson Correlation Coefficient (r)
1
0.8
0.6
0.4
0.2
0
-0.2
-0.4
-0.6
-0.8
-1

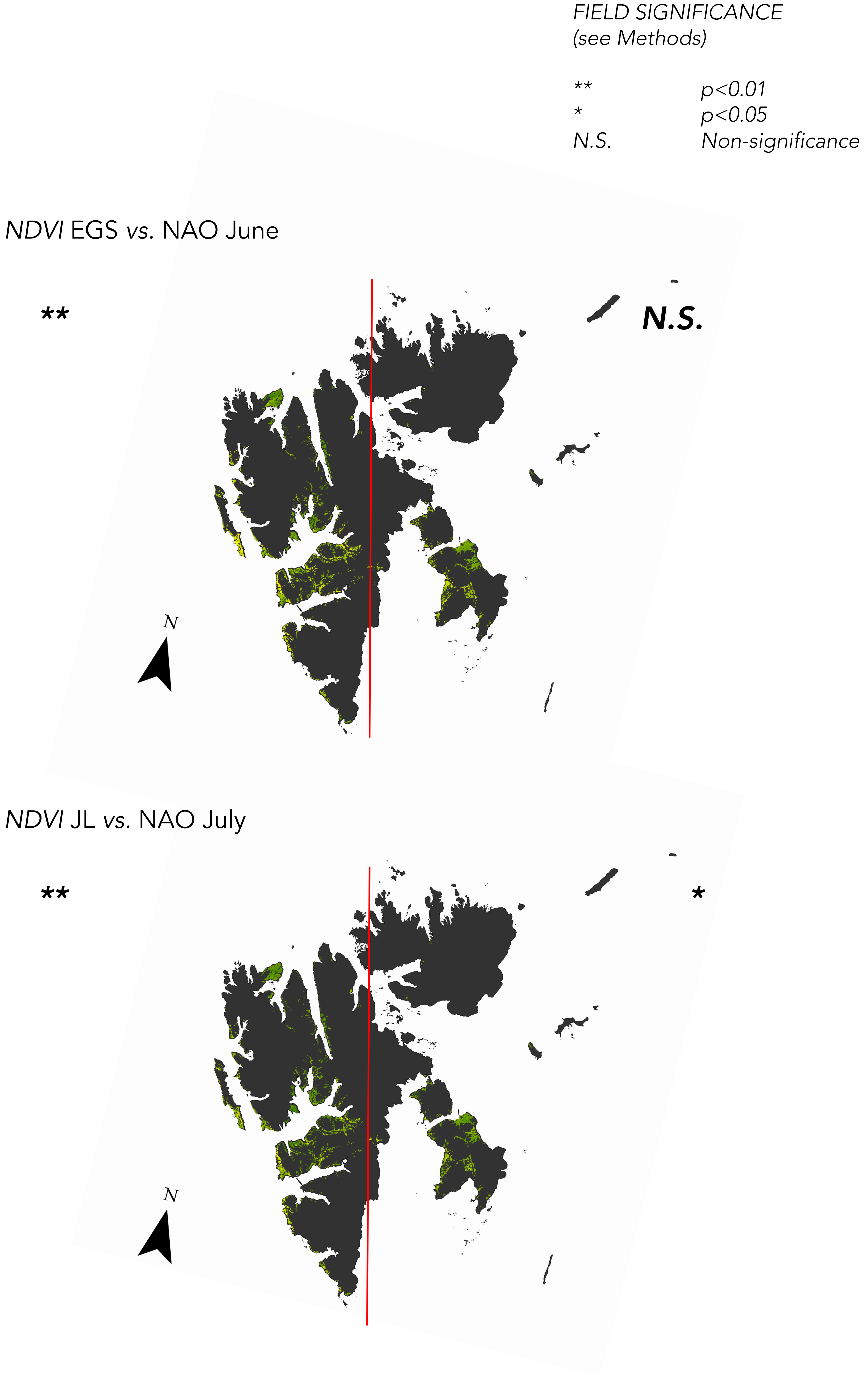
FIELD SIGNIFICANCE
(see Methods)
** p<0.01
* p<0.05
N.S. Non-significance
NDVI EGS vs. NAO June
**
N.S.
N
NDVI JL vs. NAO July
**
*
N

FIELD SIGNIFICANCE
(see Methods)

**	*p<0.01*
*	*p<0.05*
N.S.	*Non-significance*

NDVI LGS *vs.* NAO August

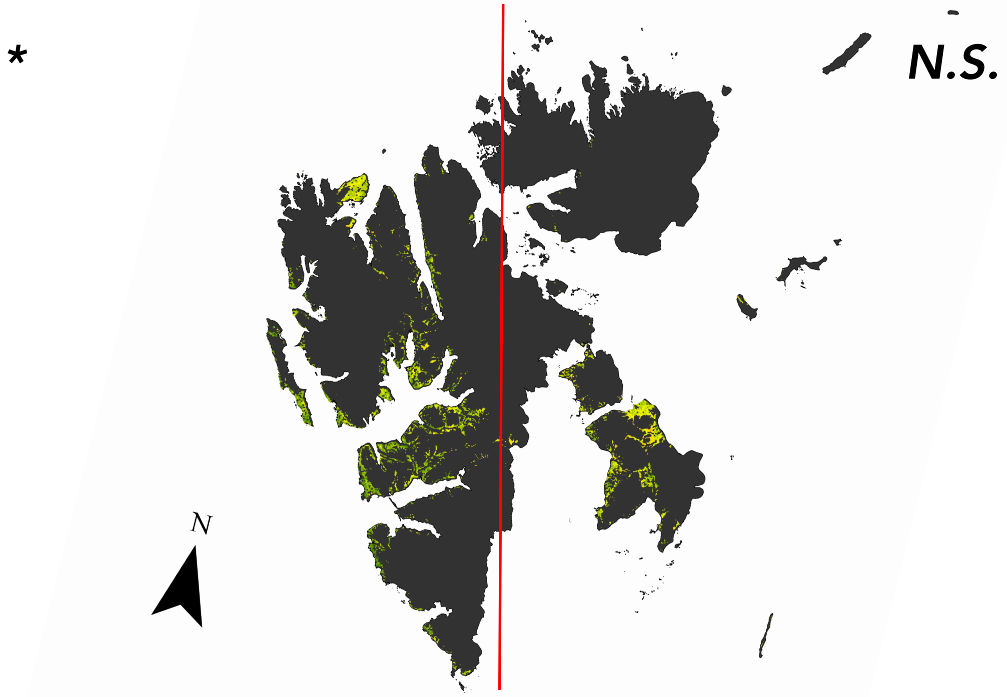

NDVI GSE *vs.* AO June

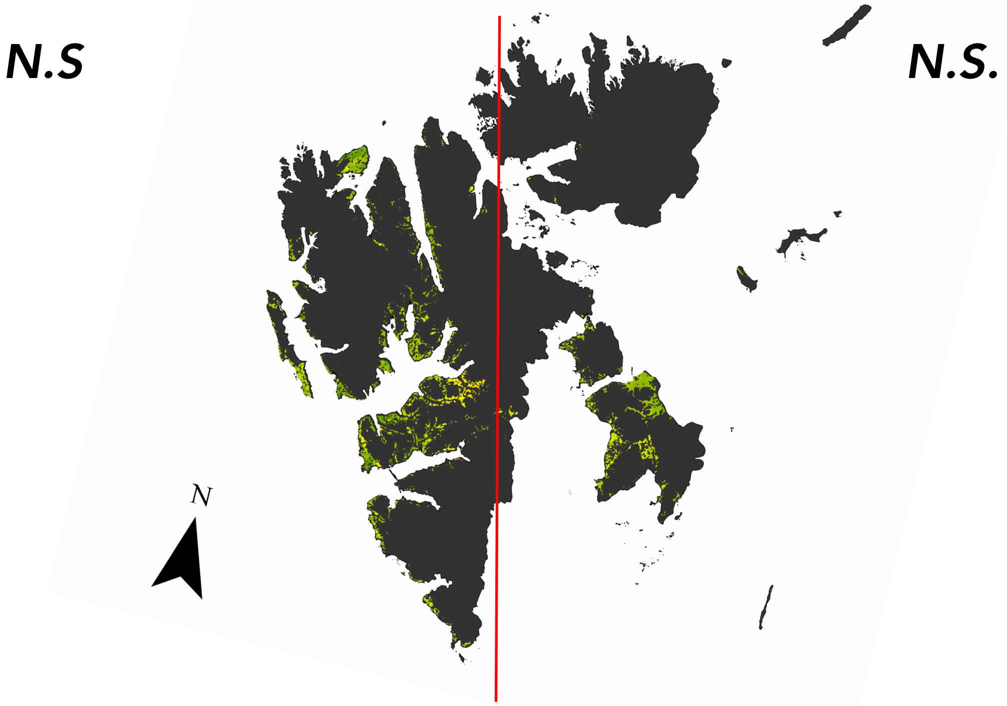

FIELD SIGNIFICANCE
(see Methods)

**	*p<0.01*
*	*p<0.05*
N.S.	*Non-significance*

NDVI JL *vs.* AO July

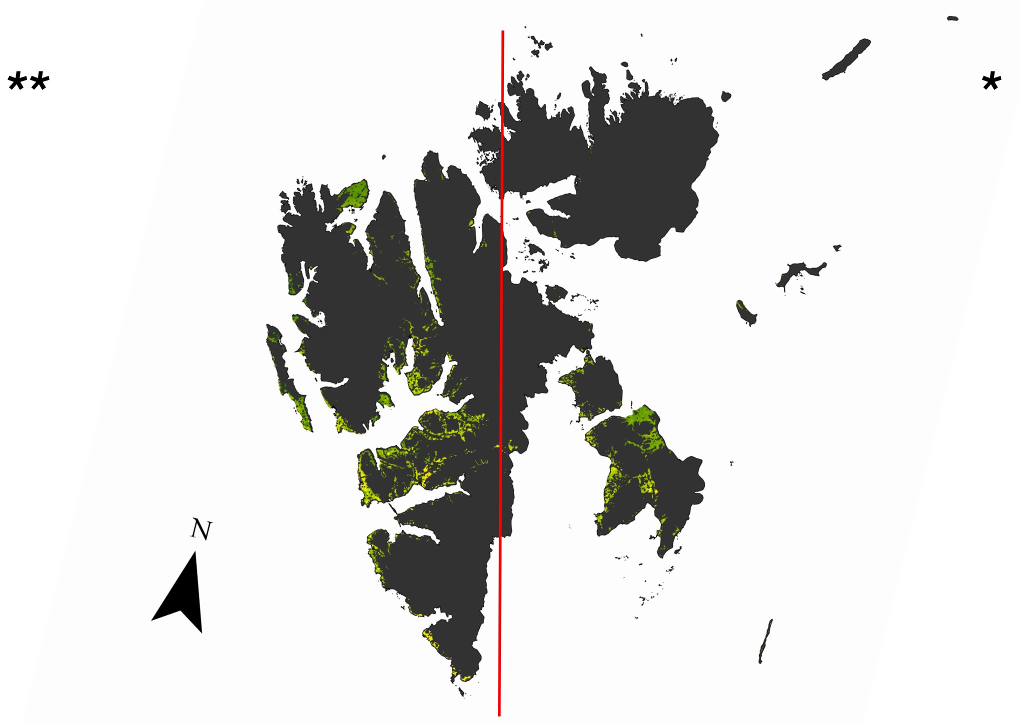

NDVI GSL *vs.* AO August

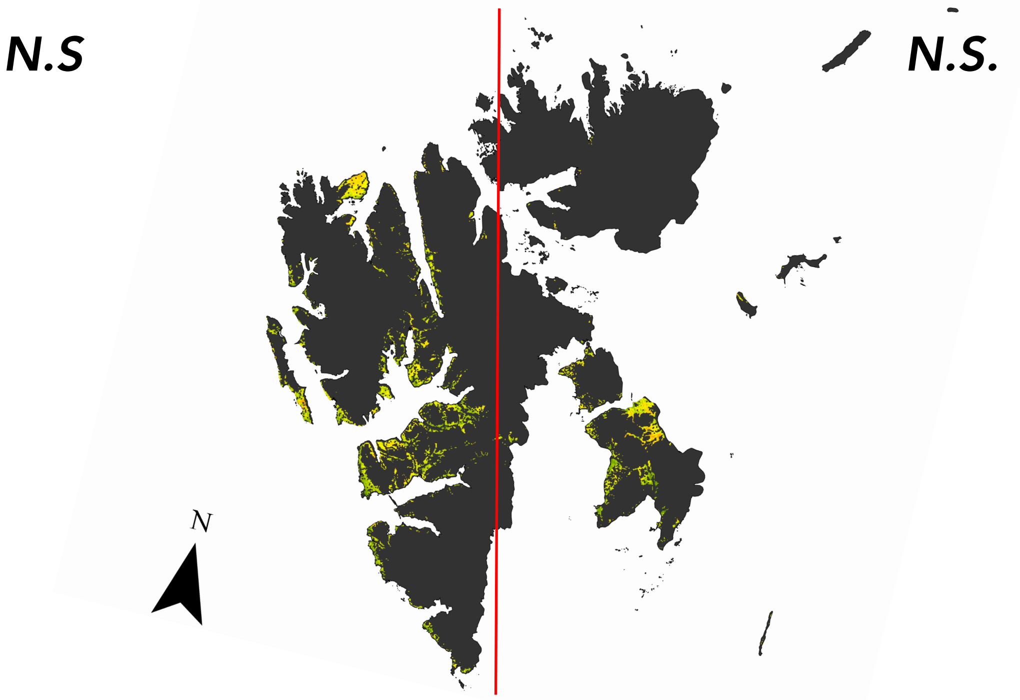

FIELD SIGNIFICANCE
(see Methods)

**	$p<0.01$
*	$p<0.05$

Field significance tables

Each significance level was computed after 1,000 time series randomly selected from a Normal distribution were generated and correlated with the fields (*NDVI* & Sea Ice concentration grids, for *W-Sb* and *E-Sb*). Grids showing very high spatial autocorrelation will require a higher % of area with significant linear Pearson coefficients for field significance.

AO and *NAO* June, July, and August monthly time series showed very low *AR1* (values ranged from 0.1 to 0.3 for different months). For n=15 years of data, and hence 13 (n-2) degrees of freedom, $p<0.05$ was set at $r=0.553$ for individual correlations from the two-tailed Student's t distribution.

Observed – NAO		*GSE*/June	JL/July	*GSL*/August
Sea Ice	*E-Sb*	0.086%	3.33%	0
	W-Sb	7.6%	23.37%**	0
NDVI	*E-Sb*	9.2%	40.66%*	2.8%
	W-Sb	23.48%**	44.66%**	13.97%*

Observed –AO		*GSE*/June	JL/July	*GSL*/August
Sea Ice	*E-Sb*	1.35%	15.41%	0.1%
	W-Sb	7.35%	8.73%*	0.16%
NDVI	*E-Sb*	0.37%	17.24%*	3.00%
	W-Sb	6.56%	24.29%**	1.09%

MC thresholds Field Significance		*E-Sb*			*W-Sb*		
		GSE June	*JL* July	*GSL* August	*GSE* June	*JL* July	*GSL* August
Sea Ice	*P<0.05*	16.53%	18.71%	12.55%	9.69%	8.71%	7.58%
	P<0.01	31.58%	31.90%	28.28%	17.22%	16.76%	15.02%
NDVI	*P<0.05*	19.62%	16.41%	10.66%	12.67%	11.14%	9.63%
	P<0.01	58.92%	58.55%	17.33%	21.87%	24.24%	16.97%

Supplementary Material 5
Reanalysis monthly correlations between AO, NAO & SAT and Meridional Wind.

Upper. Correlation fields between concurrent *Reanalysis* monthly Surface Air Temperature (*SAT*) and the North Atlantic Oscillation (*NAO*) – Period 2000-2014. Note the overall positive values, which become high in the southern half of the archipelago in July.

Lower. Correlation fields between concurrent *Reanalysis* monthly Meridional Wind (*MW*) and the *NAO*. Note the very high values in July over the western half of the region.

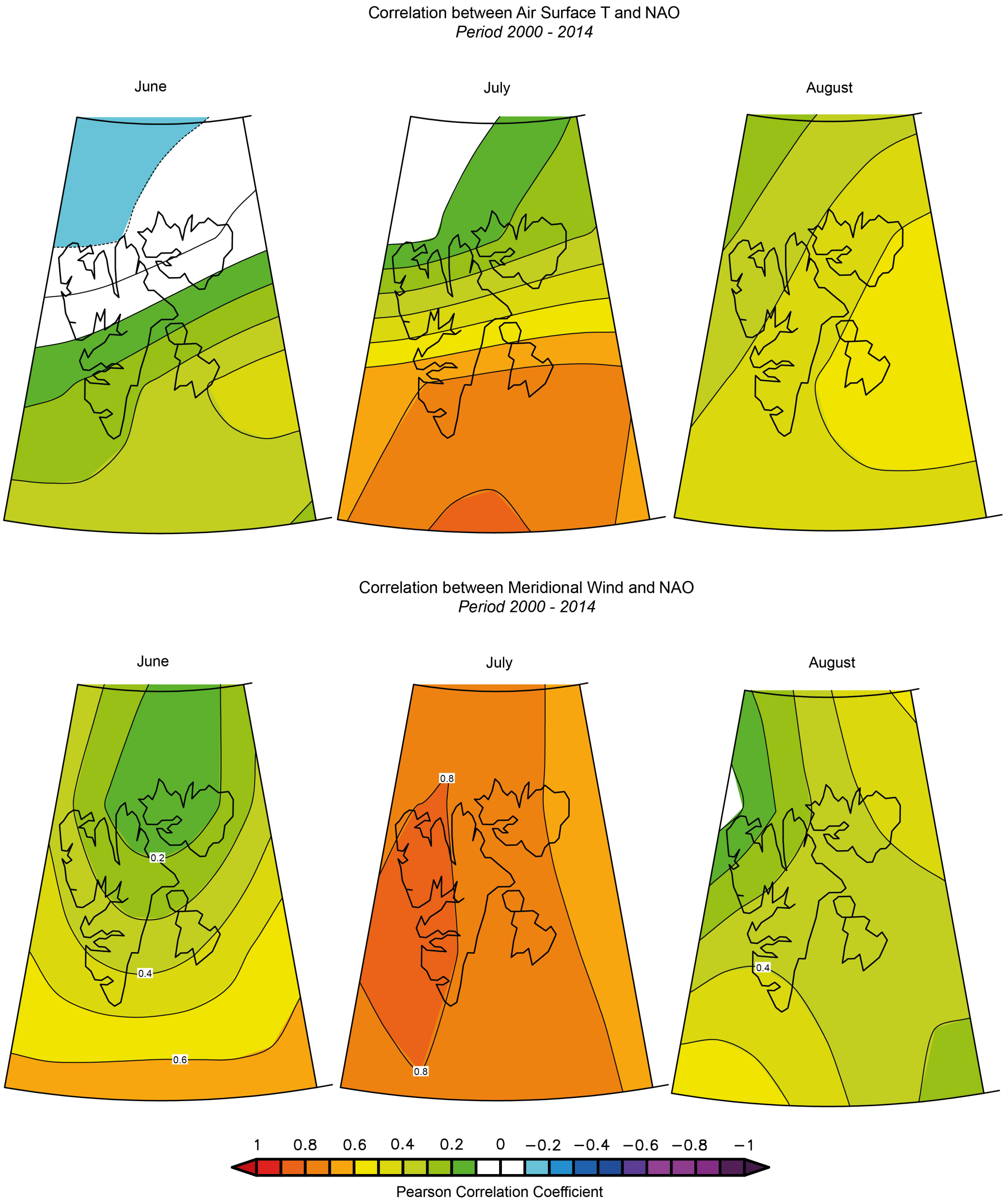

Images obtained from the NCEP-NCAR Reanalysis project [1] with permission by the NOAA/ESRL Physical Sciences Division, Boulder Colorado from their Web site at https://www.esrl.noaa.gov/psd/data/composites/reference.html

[1] Kalnay, E. *et al.* 1996, "The NCEP/NCAR 40-Year Reanalysis Project". *Bulletin of the American Meteorological Society* **77**, 437-471, doi:10.1175/1520-0477

Upper. Correlation fields between concurrent *Reanalysis* monthly *SAT* and the Arctic Oscillation (*AO*) – Period 2000-2014. Positive values only become widespread in the southern half of the archipelago in July, albeit with overall low values.

Lower. Correlation fields between concurrent *Reanalysis* monthly *MW* and the *AO*. Although positive values dominate during June and July, correlations are overall weak, and reverse in August.

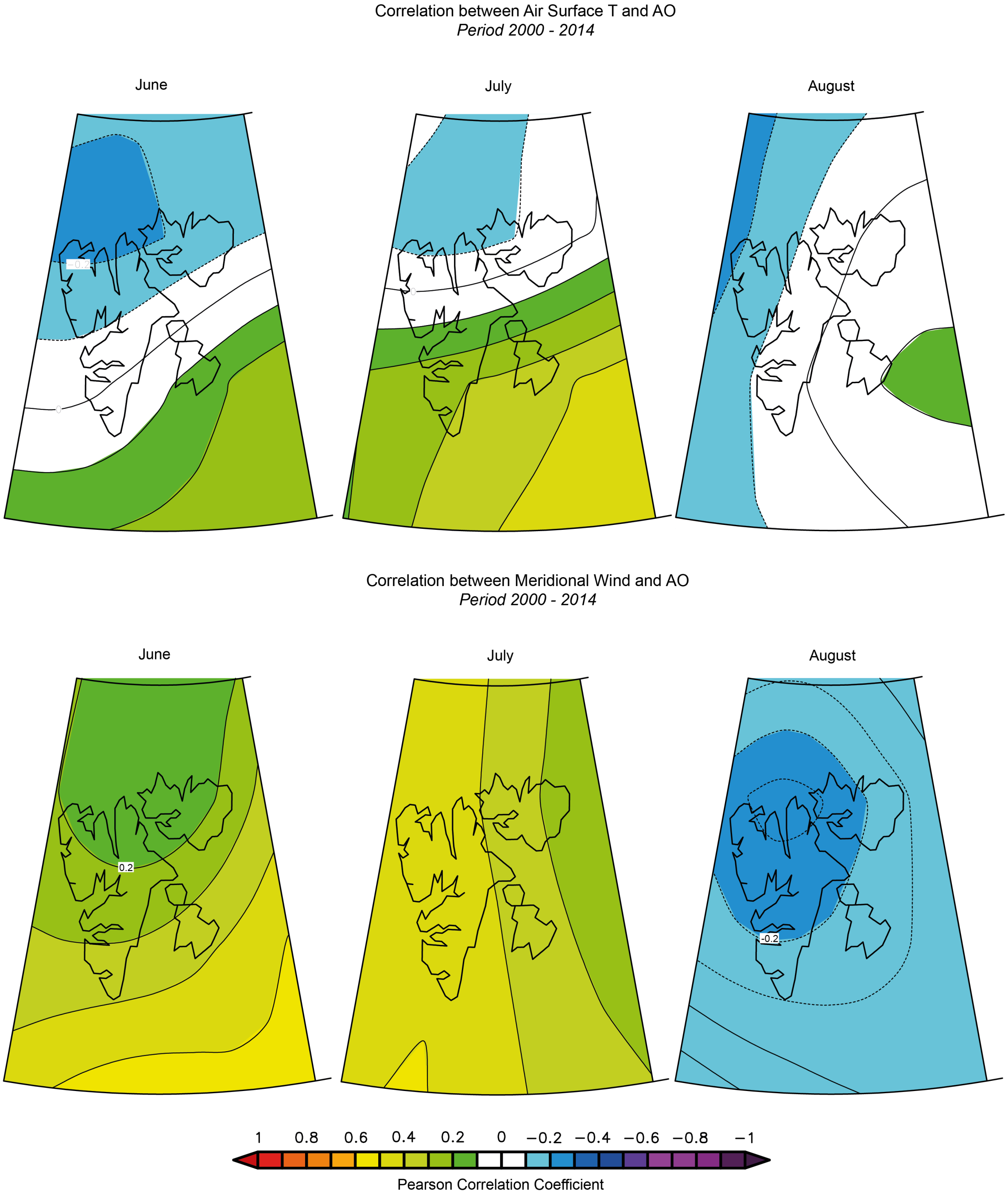

Supplementary Material 6
Local Air Temperature variability during the growing season.

Local Air Temperature variability for 6 meteorological stations located in *W-Sb* (**left**) & 6 meteorological stations located in *E-Sb* (**right**), for the period for which daily data was available in all stations (2012-2014). See map in ***Supplementary Material 1*** for their location and station code. Daily values were obtained from http://eklima.met.no. 8-day mean values (plotted) were computed when vales for ≥ 3 days were available during each 8-day period (in the immense majority of cases daily values were available for 8 out of the 8 days within the study period, except for the pre-growing season 2012 in *Karl XII-Øya*). Note the overall colder temperatures in *E-Sb*. For each station, standard deviation (*std*) values for each 8-day period across the 3 years were higher in *E-Sb* (larger inter-annual variability) than in *W-Sb* stations: the minimum *std* reported in *W-Sb* (1.31, Hopen) is higher than the maximum *std* reported in *W-Sb* (Seagruva, 1.27); std_{mean} *E-Sb* = 1.62; std_{mean} *W-Sb* = 1.18. *F-test* statistics showed significant differences in the variance between the two regions during the *GS* (green box, p<0.001) and non-significant differences in the variance out of the growing season.

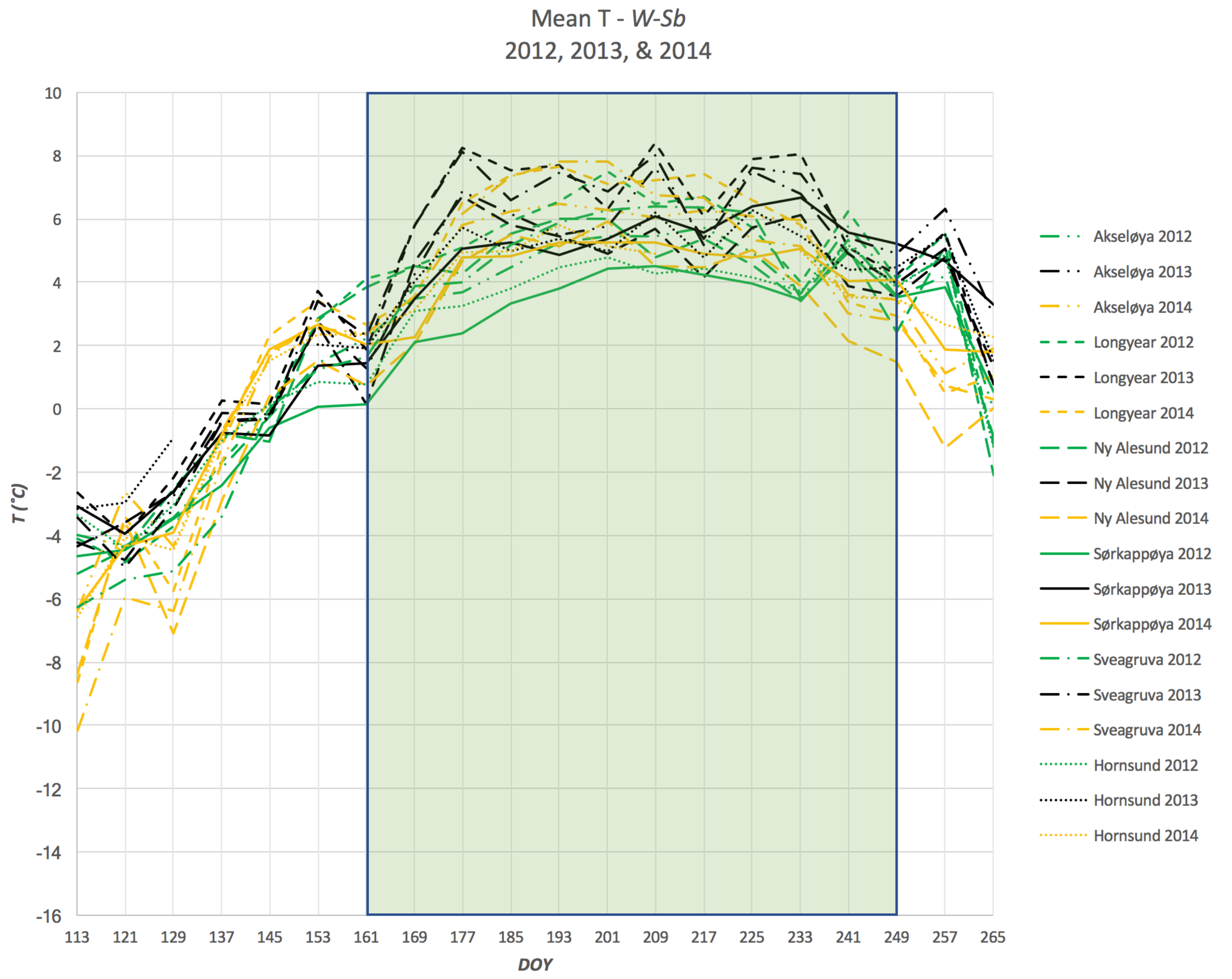

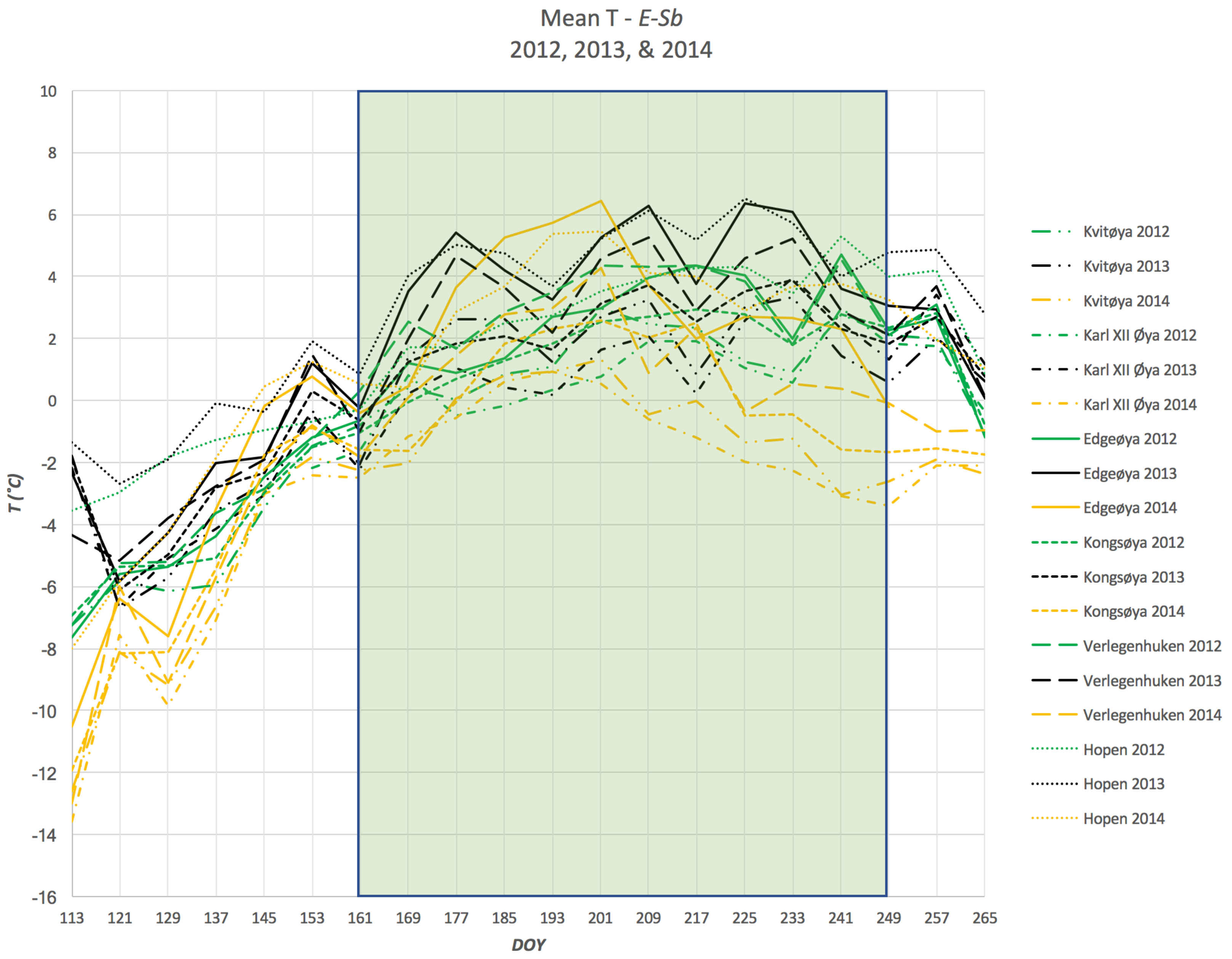

Supplement: Supplementary file 1 — Supplementary Information [file 41598_2017_6218_MOESM1_ESM.pdf]
